# MDMA alters fear extinction, and reduces alcohol consumption in inbred alcohol preferring iP rats but not outbred Wistar rats

**DOI:** 10.1038/s41386-026-02394-2

**Published:** 2026-03-27

**Authors:** Kade L. Huckstep, Billi Newton, Grace Bailey, Annai Charlton, Amy J. Pearl, Xavier J. Maddern, Robyn M. Brown, Gavan P. McNally, Dan I. Lubman, Shalini Arunogiri, Kirsten C. Morley, Erin J. Campbell, Andrew J. Lawrence, Leigh C. Walker

**Affiliations:** 1 Florey Institute of Neuroscience and Mental Health, Melbourne, VIC Australia; 2 Florey Department of Neuroscience and Mental Health, University of Melbourne, Melbourne, VIC Australia; 3 Biochemistry and Pharmacology Department, University of Melbourne, Melbourne, VIC Australia; 4 School of Psychology, University of New South Wales, Sydney, NSW Australia; 5 Turning Point, Eastern Health, Melbourne, VIC Australia; 6 Eastern Health Clinical School, Faculty of Medicine, Nursing and Health Sciences, Monash University, Melbourne, VIC Australia; 7 Sydney Medical School, Faculty of Medicine and Health, University of Sydney, Sydney, NSW Australia; 8 School of Biomedical Sciences and Pharmacy, College of Health and Medicine and Wellbeing, University of Newcastle, Callaghan, NSW Australia; 9 Brain Health Neuromodulation Research Program, Hunter Medical Research Institute, New Lambton Heights, Newcastle, NSW Australia

**Keywords:** Experimental models of disease, Stress and resilience

## Abstract

Comorbidity between post-traumatic stress disorder (PTSD) and alcohol use is common and mutually-reinforcing, yet there are no pharmacological strategies that specifically target trauma-linked escalation of alcohol intake. We evaluated whether 3,4-methylenedioxymethamphetamine (MDMA), given in a therapy-adjunctive fashion (30 min before fear extinction), could facilitate extinction of conditioned fear and reduce alcohol consumption in a rat model that combines fear conditioning, binge-like alcohol access, abstinence, and re-exposure. Inbred alcohol-preferring (iP) and outbred Wistar rats of both sexes underwent auditory fear conditioning, voluntary ethanol drinking, and subsequently fear extinction after MDMA or vehicle administration, with drug-free extinction recall and alcohol consumption assessed thereafter. Fear conditioning increased voluntary alcohol intake only in iP rats, suggesting a genotype-related fear–alcohol contingency. MDMA acutely reduced freezing during extinction, but ultimately reshaped across-session freezing patterns in a strain- and sex-dependent manner. There were no lasting MDMA treatment effects on next-day drug-free recall. MDMA also altered on-drug fear-expression during extinction without affecting later recall in an iP rat cohort without prior alcohol exposure, indicating the effect is not secondary to drinking history. Critically, MDMA prevented the shock-related increase in alcohol consumption but only in iP rats. These data suggest MDMA’s most reliable action in this model is to disrupt trauma-linked escalation of alcohol intake in genetically- and experientially- vulnerable rats, rather than to globally enhance fear extinction.

## Introduction

Post-Traumatic Stress Disorder (PTSD) and Alcohol Use Disorder (AUD) frequently co-occur and present greater symptom severity, poorer treatment response, and higher relapse risk than either alone [1–3]. One explanation for this comorbidity is the self-medication hypothesis, whereby alcohol use serves to alleviate distressing trauma-related symptoms [4, 5]. Longitudinal studies demonstrate that AUD often follows trauma exposure [5–7], and many individuals report using alcohol to manage hyperarousal, intrusive memories, or negative affect [8]. While drinking may temporarily relieve PTSD symptoms, alcohol intake dysregulates stress systems [9, 10], exacerbating PTSD symptoms and maintaining the cycle of use and relapse [1, 10]. Not all trauma-exposed individuals develop PTSD, nor all drinkers AUD, with only a subset experiencing both, highlighting heterogeneity [11]. Shared genetic vulnerabilities and overlapping neurobiological mechanisms, particularly stress and reward-circuit dysregulation contribute to comorbidity [12, 13] and may outline clinically-relevant subgroups in whom trauma-related distress more strongly motivates alcohol seeking [3, 12]. No medications are approved specifically for this dual diagnosis [2, 3], and meta-analyses show limited efficacy of sequential treatment [2, 14]. This underscores the need for integrated, mechanism-informed approaches that simultaneously address both PTSD and alcohol consumption.

3,4-methylenedioxymethamphetamine (MDMA) has re-emerged as a promising adjunct to psychotherapy for PTSD. Phase III clinical trials report durable reductions in PTSD symptoms [15–18], with emerging evidence suggesting secondary reductions in hazardous alcohol use [19] although participants did not meet AUD criteria. An open-label AUD pilot study found MDMA-assisted therapy to be safe, well tolerated, and associated with reduced post-detoxification drinking [20, 21]. However, these findings were limited by small sample size, retrospective self-report, and a lack of control conditions. Furthermore, expectancy and blinding challenges complicate the interpretation of clinical outcomes [22, 23], and questions remain about relapse risk in AUD populations [24] and whether alcohol effects depend on being embedded in trauma-focused procedures. Thus, before assuming MDMA will simultaneously improve PTSD and alcohol outcomes, it is necessary to adopt a model that couples traumatic stress-exposure with alcohol consumption and determine whether MDMA given primarily to address PTSD-like symptoms also alters relapse-like drinking.

PTSD involves maladaptive learning and memory, including persistent threat [11, 25, 26]. Pavlovian fear conditioning models aetiological and treatment-relevant learning processes: neutral cues can become associated with trauma, while extinction establishes a competing inhibitory “safety” memory [27]. This underlies first-line evidence-based exposure-based psychotherapies for PTSD [28], yet PTSD patients also exhibit deficient extinction learning, retrieval, and/or expression [26, 29]. Given this pathological persistence, and impaired ability to extinguish conditioned fear, MDMA’s promising clinical results are potentially explained by enhanced learning and/or retention of fear extinction [30]. In patients whose alcohol use is functionally tied to PTSD-related distress, improving fear extinction fear could, in principle, also reduce motivation for alcohol [19, 20].

MDMA can facilitate fear extinction in healthy humans, albeit with some heterogeneity [31, 32]. Rodent studies similarly suggest that MDMA can enhance extinction learning and recall [33, 34] with mixed findings relating to species/strain [35]. MDMA can also reduce alcohol consumption and preference in rats [36], but no studies have assessed the effect of pairing MDMA with extinction in a model that captures the functional PTSD-alcohol use co-occurrence – that is, where trauma-like cues, extinction, and alcohol consumption are all present.

Here we address this gap by combining Pavlovian fear conditioning with voluntary alcohol access in inbred Indiana alcohol-preferring (iP) rats and outbred Wistar rats. MDMA was administered immediately before extinction to mimic therapy-adjunctive dosing, and we quantified acute fear expression, extinction recall, and subsequent alcohol intake. We hypothesised that MDMA would enhance extinction learning/recall and reduce alcohol consumption, with the strongest effects in iP rats.

## Methods

**Fig. 1:**
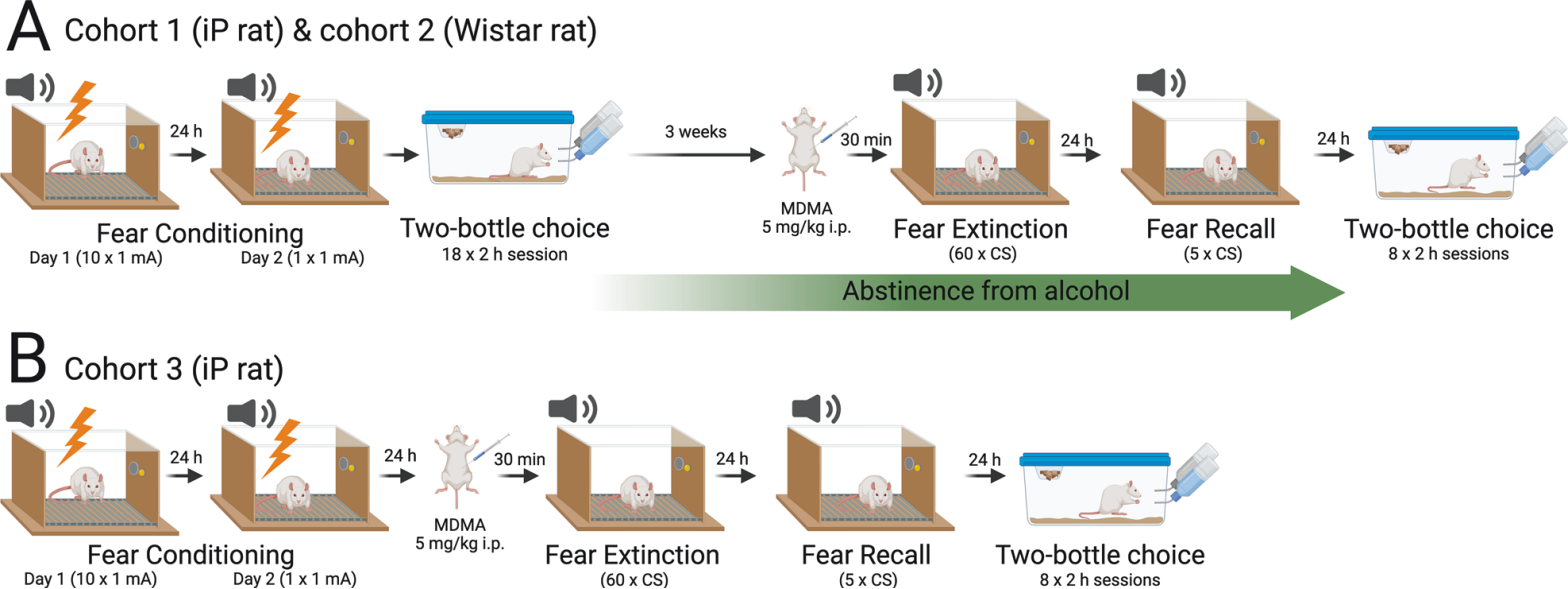
Schematic outline of experimental timeline for cohort 1 & 2. **A** To assess the effects of MDMA on fear extinction and alcohol consumption, cohort 1 (iP rats) & cohort 2 (Wistar rats) underwent fear conditioning over 2 days, followed by access to alcohol on a limited access schedule for 6 weeks (18 session). After 3 weeks of abstinence, rats were intraperitoneally injected with MDMA (or VEH) and placed into fear extinction 30 min following this. After 24 h, when MDMA was no longer present, rats were tested for fear extinction recall, then given re-access to alcohol the following day. **B** In cohort 3, iP rats underwent a similar behavioural procedure; however, rats moved directly from fear conditioning to MDMA administration and fear extinction 24 h later. Following extinction, rats underwent fear recall and two-bottle choice alcohol access as per cohort 1 & 2. Figure created with Biorender (L Walker, MU29CIVU7T).

Alcohol-preferring iP rats (*N* = 78) and Wistar rats (*N* = 64) were used in studies. All experiments were conducted in accordance with the Prevention of Cruelty to Animals Act, National Health and Medical Research Council (NHMRC) Australian Code of Practice and approved by The Florey Animals Ethics Committee. Full methods are described in the supplemental materials and detailed timelines shown in Fig. 1.

Cohort 1, alcohol-preferring iP rats (female=28, male=25), and cohort 2 (Wistar rats, female=32, male=32) underwent 2 days of fear conditioning. On Day 1, rats were presented the conditioned stimulus tone (CS) for 10 s, co-terminating with a 1 s footshock (unconditioned stimulus; US, 1 mA). Rats received 10 CS-US (shock + tone) pairings. No shock control rats underwent the same protocol; however, they received the tone alone. On Day 2, shock rats received one CS-US pairing. Rats from cohorts 1 and 2 were then given voluntary access to 20% v/v ethanol or water in a two-bottle, intermittent modified drinking in the dark procedure [37–39]. Rats underwent 18 ×2 h binge drinking sessions over 6 weeks. Following this, rats were placed into abstinence for 3 weeks. To assess whether MDMA alters fear extinction, freezing in response to the CS was assessed 30 min after acute MDMA (5 mg/kg, i.p) or vehicle (saline) administration. To assess enduring effects, fear recall was assessed 24 h after MDMA administration (5 CS presentations). After 24 h, rats were given free access to alcohol in a two-bottle choice protocol to assess post-treatment alcohol intake and continued drinking for 8 sessions (Fig. 1A).

Finally, to assess the temporal dynamics and effect of prior alcohol consumption on MDMA-elicited fear responses, another cohort of iP rats (female=9, male=16), underwent the same protocol as cohort 1 and 2, however, they were administered MDMA (5 mg/kg, i.p) or saline prior to alcohol exposure and underwent fear extinction 24 h after day 2 fear conditioning (Fig. 1B). All statistical analysis was performed using GraphPad Prism 10, with significance set at *p* < 0.05. All data figures are represented as mean ± SEM, full statistics are reported in Supplementary Table S1. Where no effect of sex was observed, data were reanalysed to pool sexes together, where sex effects were observed further sex separated analysis were conducted and provided in Figs. S2 and S4.

## Results

### Fear conditioning increases alcohol consumption in inbred iP rats, but not outbred Wistar rats

**Fig. 2:**
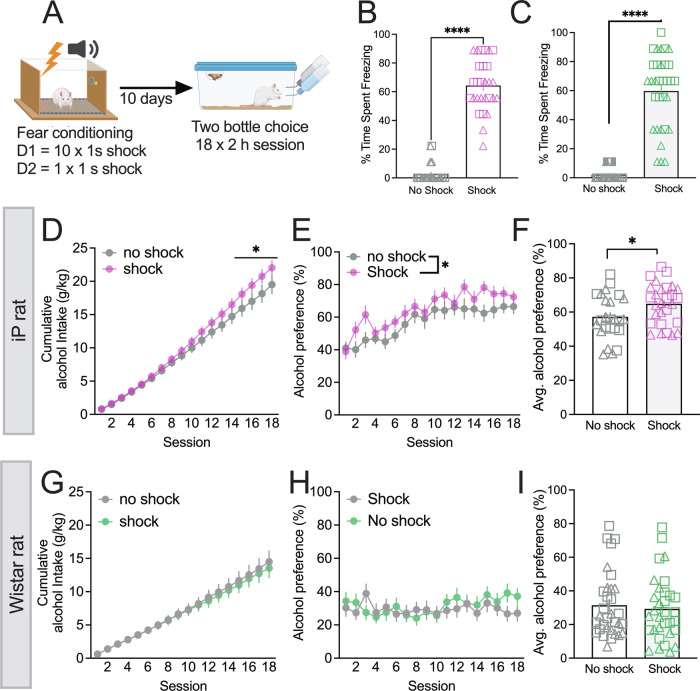
Fear conditioning increases alcohol consumption in iP, but not Wistar rats. **A** Schematic overview. **B** Alcohol preferring iP rats and **C** Wistar rats that underwent fear conditioning on day 1 showed increased % time freezing during CS presentation on day 2 compared to no shock controls, however male Wistar rats show greater freezing than females (see Fig. S1). **D** iP rats exposed to footshock showed a subsequent increase in cumulative alcohol consumption across two-bottle choice sessions and increased preference for alcohol **E** across sessions, and **F** average preference compared to no shock controls. **G** Wistar rats exposed to footshock showed no change in cumulative alcohol consumption, or **H** preference across two-bottle choice sessions, or in **I** average preference compared to no shock controls. Please see Fig. S1 for analysis of Wistar rats showing sex differences. Female triangle symbols, male square symbols. Data analysed by unpaired *t*-test **B, F, I** or two-way **C, D, E, G, H** or three-way **H** ANOVA with Bonferroni post-hoc, *p < 0.05, ***p < 0.001, ****p < 0.0001. Data represented as mean ± SEM.

Given the established bidirectional relationship between PTSD and alcohol use [40, 41], we first assessed fear conditioning, and its impact on subsequent alcohol consumption in male and female rats. No effect of sex was observed across behaviour in iP rats, therefore sex was not included as a variable in analyses (See Table S1). In iP rats, an unpaired t-test revealed greater percentage of time spent freezing following footshock (t = 16.17, df=51, *p* < 0.0001; Fig. 2B). In Wistar rats, footshock also elicited a freezing response (main effect shock F_(1,60)_ = 187.5, *p* < 0.0001), however, a main effect of sex (F_(1,60)_ = 7.868, *p* = 0.0068), and a sex x shock interaction (Sex x Shock = F_(1,60)_ = 4.601, *p* = 0.0360) was observed. *Post-hoc* analysis revealed both male and female Wistar rats increased freezing after footshock (p’s<0.0001), but males showed greater freezing than female rats (*p* = 0.0009, Fig. 2C, Fig. S2A, B).

No sex differences in alcohol consumption or preference were observed in iP rats (see Table S1). However, footshock showed a modest, but reliable enhancement of cumulative alcohol intake over time (main interaction shock x session F_(17,863)_ = 2.765, *p* = 0.0002), with *post-hoc* analysis showing difference during session 15–18 compared to no shock controls (p’s <0.05, Fig. 2D). A main effect of shock was also observed on alcohol preference with iP rats exposed to footshock showing higher sessional alcohol preference (main effect Shock F_(1,51)_ = 5.045, *p* = 0.0291, Fig. 2E) and average preference across the study (t = 2.242, df=51, *p* = 0.0294).

In contrast, no effect of footshock was observed in Wistar rats for cumulative intake (F_(1,62)_ = 0.06373, *p* = 0.8015, Fig. 2G), preference across session (F_(1,60)_ = 0.1973, *p* = 0.6585, Fig. 2H) or average alcohol preference (F_(1,60)_ = 0.1981, *p* = 0.6579, Fig. 2I). However, some sex differences were observed (detailed in Table S1 and Fig. S1). Comparisons between rat strains showed no difference between iP and Wistar rats in fear conditioning (main effect strain F_(1,113)_ = 0.7141, *p* = 0.3999, Fig. S2A), but iP rats consumed more alcohol (main effect strain F_(1,63)_ = 16.73, *p* = 0.0001, Fig. S2B) and had higher alcohol preference (main effect strain F_(1,62)_ = 113.9, *p* < 0.0001, Fig. S2C) than Wistar rats.

### MDMA alters fear extinction, but not recall in iP and Wistar rats

#### Fear extinction

**Fig. 3:**
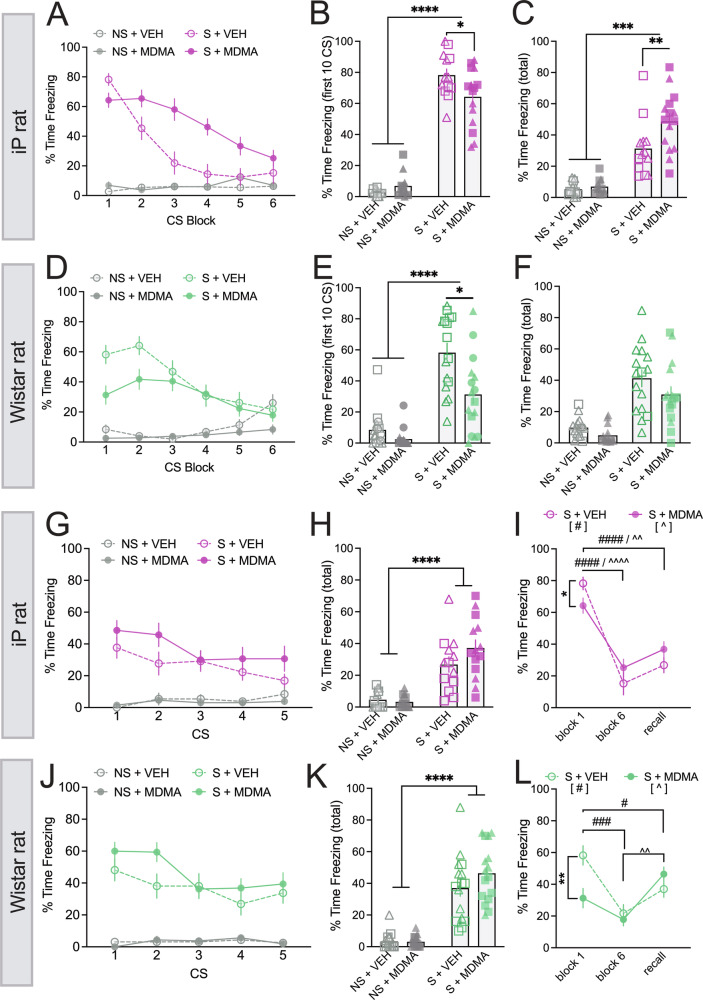
MDMA alters fear extinction but not recall in iP rats and Wistar rats. **A** MDMA altered % time spent freezing during CS presentation across extinction trials in iP rats **B** In the first CS block (10 CS’s), shock-exposed iP rats spent more % time freezing than no-shock controls, with those who received MDMA freezing significantly less than those who received vehicle. **C** iP rats exposed to shock also spent more time freezing (than no-shock controls) over the total extinction session, however, MDMA-treatment instead increased the total % time spent freezing. **D** MDMA altered % time spent freezing during CS presentation in Wistar rats across time. **E** In both the first CS block and **F** in the total test, shock-experienced Wistar rats spent more % time freezing than no shock rats, however this was reduced in shock rats that received MDMA but driven by female rats (see Fig. S2). **G** iP rats showed no difference in fear extinction recall freezing across CS or **H** in total. **I** Changes in freezing behaviour from CS block 1 to block 6 and recall 24 h later. **J** Wistar rats showed no difference in fear recall across CS or **K** in total % time freezing. **L** Changes in freezing behaviour from CS block 1 to CS block 6 and recall 24 h later. Female triangle symbols, male square symbols. Data analysed by two, or three-way ANOVA with Bonferroni post-hoc, ****p < 0.0001, ***p < 0.001, **p < 0.01, *p < 0.05. In **I** and **L**: * denotes significant difference between treatment groups at discrete timepoints, # denotes significant effect between timepoints within VEH group, ^ denotes significance between timepoints within MDMA group. Data represented as mean ± SEM. N no shock, S shock.

MDMA has shown clinical promise in the treatment of PTSD and is thought to act by enhancing fear extinction [33, 34]. Therefore, we next assessed how MDMA influences fear extinction. In iP rats, three-way ANOVA of freezing behaviour revealed main effects of Shock (F_(1,49)_ = 79.57, *p* < 0.0001); CS block (F_(5,245)_ = 33.85, *p* < 0.0001); Treatment (F_(1,49)_ = 6.315, *p* = 0.0153), and interactions of CS block x Shock (F_(5,245)_ = 42.84, *p* < 0.0001); CS block x Treatment (F_(5,245)_ = 6.833, *p* < 0.0001); Shock x Treatment; (F_(1,49)_ = 4.328, *p* = 0.0427) and CS block x Treatment x Shock (F_(5,245)_ = 9.126, *p* < 0.0001; Fig. 3A). Analysis assessing % time freezing in the first 10 CS showed no effect of Sex. Two-way ANOVA followed by Bonferroni *post-hoc* analysis showed increased freezing in VEH shock and MDMA shock compared to no shock controls (p’s<0.0001). Further, the MDMA shock group showed decreased freezing compared VEH shock iP rats (*p* = 0.0229; Fig. 3B). Analysis of total % time CS freezing showed no effect of sex. Two-way ANOVA followed by Bonferroni *post-hoc* analysis showed greater total freezing in VEH shock and MDMA shock iP rats compared to no shock iP rats (p’s<0.0001). An increase in freezing was observed in MDMA shock rats compared to VEH shock rats (*p* = 0.0064; Fig. 3C). Together, MDMA enhanced early extinction in iP rats but also impaired overall within-session extinction.

In Wistar rats, three-way ANOVA revealed main effects of Shock (F_(1,60)_ = 57.41, p < 0.0001), CS block (F_(5,300)_ = 8.844, *p* < 0.0001), and Treatment (F_(1,60)_ = 4.038, *p* = 0.0490), and interactions between Shock x CS block (F_(5,300)_ = 33.59, *p* < 0.0001), CS block x Treatment (F_(5,300)_ = 4.265, *p* = 0.0009), and CS block x Shock x Treatment (F_(5,300)_ = 5.324, *p* < 0.0001; Fig. 3D). Three-way ANOVA (Shock/Treatment/Sex) analysis assessing % time freezing in the first 10 CS (block 1), showed a main effect of Sex (F_(1,56)_ = 4.485, *p* = 0.0387), Shock (F_(1,56)_ = 78.15, *p* < 0.0001); Treatment (F_(1,56)_ = 13.66, *p* = 0.0005); and Shock x Treatment interaction (F_(1,56)_ = 5.625, *p* = 0.0212). Bonferroni *post-hoc* analysis showed greater freezing in VEH shock and MDMA shock groups of both sexes compared to controls (p’s<0.005; Fig. 3E). Further analysis by sex showed shock MDMA females exhibited decreased freezing compared to VEH shock females (*p* = 0.005), but no difference was observed in males (VEH shock vs. MDMA shock *p* > 0.9999; Fig. S1C-D). Analysis of total % time freezing showed a main effect of Sex (F_(1,56)_ = 11.19, *p* = 0.0015), Shock (F_(1,56)_ = 74.39, *p* < 0.0001), Treatment (F_(1,56)_ = 5.232, *p* = 0.0260), and a Shock x Sex interaction (F_(1,56)_ = 7.687, *p* = 0.0075). Bonferroni *post-hoc* analysis showed VEH shock female rats froze more than VEH no shock females (*p* < 0.001), however VEH shock males did not differ from VEH no shock males (*p* = 0.1088). Additionally, VEH shock female rats froze more than MDMA shock female rats (*p* = 0.005, Fig. 3F & Fig. S1E, F). Thus, in Wistar rats, MDMA enhanced both early and overall, within session extinction, an effect predominantly driven by females (see Fig. S1 for analyses separated by sex). Importantly no difference in baseline motion levels were observed in either strain following MDMA administration, measured during the pre-CS period (Fig. S3A, B).

#### Recall of fear extinction

To assess if there were any lasting effects of MDMA on fear extinction learning, we tested drug-free fear extinction recall 24 h following extinction. In iP rats, three-way ANOVA revealed a main effect of Shock (F_(1,49)_ = 56.98, *p* < 0.0001), and CS (F_(4,196)_ = 3.163, *p* = 0.0151), and a Shock x CS interaction (F_(4,196)_ = 6.578, *p* < 0.0001), but no main effect of treatment, nor any other interactions (Fig. 3G). For total % time spent freezing no main effect of sex was observed; two-way ANOVA collapsing sex showed a main effect of Shock (F_(1,49)_ = 56.98, *p* < 0.0001), but no effect of Treatment, or Shock x Treatment interaction (Fig. 3H). Comparing freezing across extinction block 1, block 6 and recall found no main effect of Sex. Two-Way RM ANOVA revealed a main effect of Time (F_(1.738, 45.18)_ = 55.16, *p* < 0.0001) and a Time x Treatment interaction (F_(1.738,45.18)_ = 3.679, *p* = 0.0388; Fig. 3I). *Post-hoc* analysis confirmed higher freezing in VEH compared MDMA iP rats during extinction block 1 (*p* = 0.033) but no difference between treatment groups at block 6 or recall. Compared with block 1, there was less freezing at block 6 in both VEH (*p* < 0.0001) and MDMA-treated rats (*p* < 0.0001), as well as at recall (VEH *p* < 0.0001; MDMA *p* = 0.0027). Suggesting, MDMA affected within-session extinction in iP rats but there was no further effect on drug free recall.

In Wistar rats, three-way ANOVA revealed a main effect of Shock (F_(1,60)_ = 118.5, *p* < 0.0001), CS (F_(4,240)_ = 5.604, *p* = 0.0003) and a Shock x CS interaction (F_(4,240)_ = 7.890, *p* < 0.0001), but no effect of Treatment, or other interactions (Fig. 3J). Three-way ANOVA assessing total % time spent freezing showed a main effect of Sex (F_(1,56)_ = 10.02, *p* = 0.0025), Shock (F_(1,56)_ = 141.3, *p* < 0.0001), and a Shock x Sex interaction (F_(1,56)_ = 5.544, *p* = 0.0221), but no main effect of treatment, or other interactions (Fig. 3K). Bonferroni *post-hoc* analysis of sex-specific effects in Wistar rats showed footshock increased across-session freezing in females (*p* = 0.019) and males (*p* < 0.0001); this was not altered by MDMA (female *p* = 0.5865; male *p* = 0.6564; Fig. S1G-H). Comparing freezing across extinction block 1, block 6 and recall found no main effect of sex. Two-Way RM ANOVA revealed a main effect of Time (F_(1.869,56.08)_ = 13.75, *p* < 0.0001) and a Time x Treatment interaction (F_(1.869,56.08)_ = 6.245, *p* = 0.0043) but not Treatment (Fig. 3L). *Post-hoc* analysis confirmed more freezing in VEH than MDMA rats during extinction block 1 (*p* = 0.004) but no difference between treatment groups at block 6 or recall. Compared with extinction block 1, VEH animas froze less at block 6 (*p* = 0.0003) and recall (*p* = 0.0343), but not in MDMA-treated rats. The only return of fear (freezing) at recall compared with extinction block 6 was in MDMA-treated Wistar rats (*p* = 0.0048). Thus, MDMA reduced early extinction freezing in Wistars but did not improve within or between session extinction.

When comparing rat strains, iP rats exhibited more freezing in response to MDMA (delta freezing) compared to Wistar rats from CS block 2-5 (p’s<0.05, Fig. S2D). While both rat strains showed reduced freezing in CS block 1 following MDMA administration, no difference was observed between strains (*p* = 0.1729, Fig. S2E). iP rats showed greater freezing following MDMA (*p* = 0.0029), while Wistar rats trended towards a reduction (*p* = 0.0541) and a difference between strains was observed (iP>Wistar, *p* = 0.0004; Fig. S2F). While both strains showed a trend towards MDMA increasing freezing during recall, there was no strain difference (Fig. S2G). Overall, iP rats appear more sensitive to MDMA than Wistar rats.

### MDMA attenuates shock enhanced alcohol consumption in iP, but not Wistar rats

**Fig. 4:**
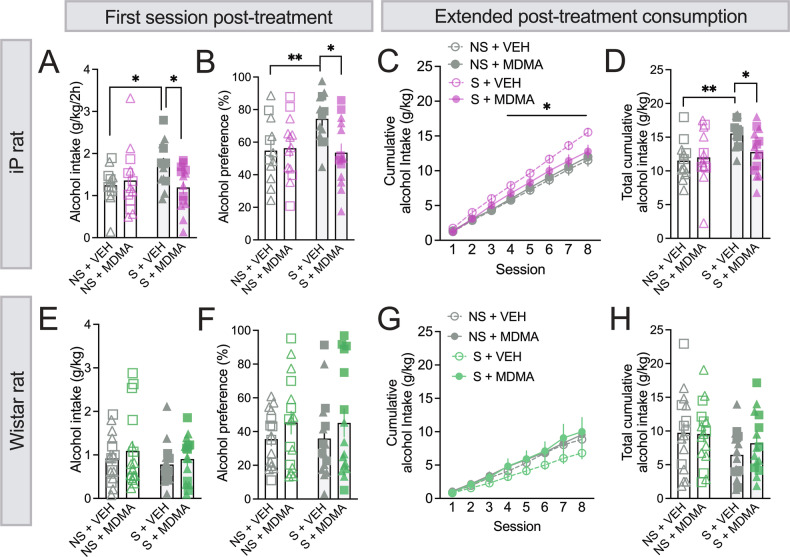
MDMA during fear extinction reduces subsequent alcohol consumption in iP, but not Wistar rats. In the first session post-treatment iP rats exhibit an **A** increase in alcohol consumption (g/kg) and **B** preference in shock + VEH rats compared to no shock + VEH rats, while shock + MDMA iP rats showed reduced alcohol consumption compared to shock + VEH controls. **C** Cumulative alcohol intake (g/kg) by session in iP rats was also higher in shock + VEH iP rats, compared to no shock + VEH rats. **D** For total consumption over 8 post-treatment sessions iP rats also showed an increase in alcohol consumption (g/kg) in shock + VEH rats compared to no shock + VEH rats, while shock + MDMA iP rats showed reduced alcohol consumption compared to shock + VEH controls. **D** In the first session post-treatment Wistar rats do not elicit shock enhanced **E** alcohol consumption, or **F** alcohol preference, nor was any impact of MDMA administration during fear extinction observed on alcohol consumption. **G** Cumulative alcohol intake (g/kg) by session and **H** total intake over 8 sessions post-treatment in Wistar rats also showed no differences based on shock or treatment, however some sex differences were observed (see Fig. S1 for analysis separated by sex). Female triangle symbols, male square symbols. Data analysed by two- or three-way ANOVA with Bonferroni post-hoc, *p < 0.05. Data represented as mean ± SEM. NS no shock, S shock.

We next determined whether MDMA during extinction learning impacted post-extinction alcohol consumption upon return to access after 3-week abstinence [42, 43]. In iP rats, no main effect of Sex was observed. Two-way ANOVA showed no main effect of Treatment, or Shock, but a Shock x Treatment interaction was observed (F_(1,48)_ = 4.120, *p* = 0.0480). Bonferroni *post-hoc* analysis showed shock VEH-treated rats increased alcohol consumption compared to no shock vehicle rats (*p* = 0.0410); however, MDMA attenuated alcohol consumption, with shock MDMA treated rats showing reduced intake compared to shock VEH rats (*p* = 0.0198; Fig. 4A). No sex effect was observed in iP rats for alcohol preference in the first session post-treatment. Two-way ANOVA showed a no main effect of Shock, but a trend towards main effect of Treatment (F_(1,48)_ = 3.498, *p* = 0.0676) and Shock x Treatment interaction was observed (F_(1,48)_ = 4.665, *p* = 0.0358; Fig. 4B). *Post-hoc* analysis showed a significant difference between VEH shock rats had increased preference compared to no shock VEH (*p* = 0.0116), and shock MDMA treated rats had reduced preference compared to shock VEH treated rats (*p* = 0.0055). Rats underwent 7 additional alcohol sessions post-treatment. Three-way ANOVA showed a main effect of Session (F_(7,336)_ = 714.8, *p* < 0.0001); Shock (F_(1,48)_ = 7.325, *p* = 0.0094), and Session x Shock interaction (F_(7, 336)_ = 6.362, *p* < 0.0001); plus a trend for a Session x Shock x Treatment interaction (F_(7,336)_ = 2.028, *p* = 0.0510). Bonferroni *post-hoc* analysis showed shock VEH consumed more alcohol than no shock VEH rats (session 3-8 p’s<0.05); however, there was no difference between MDMA no shock and shock groups (Fig. 4C). Further, total intake over the 8 sessions showed a main effect of Shock (F_(1,48)_ = 7.463, *p* = 0.0088) and trend of Shock x Treatment interaction (F_(1,48)_ = 3.411, *p* = 0.0709). Bonferroni *post-hoc* analysis showed shock VEH-treated rats had increased alcohol consumption compared to no shock vehicle rats (*p* = 0.0026); however, MDMA reduced alcohol escalation, with shock MDMA treated rats showing reduced intake compared to shock VEH rats (*p* = 0.0283; Fig. 4D) suggesting, while effect sizes were modest, in iP rats, MDMA may reduce the shock-related increases in alcohol intake.

In Wistar rats, a main effect of Sex (F_(1,56)_ = 11.59, *p* = 0.0012), but no main effect of Treatment, Shock, or any interaction was observed on alcohol consumption in the first session post-treatment (Fig. 4E). A main effect of Sex was also observed for first session post-treatment alcohol preference (F_(1,56)_ = 6.385, *p* = 0.0144) and a Sex x Treatment interaction (F_(1,56)_ = 6.707, *p* = 0.0122), but no effect of Shock, Treatment or other interactions (Fig. 4F). *Post-hoc* analysis showed no specific differences between treatment groups of either sex. Analysis of intake across post-treatment sessions showed a main effect of Session (F_(7,413)_ = 123.1, *p* < 0.0001), but no effect of Shock, Treatment, or interactions (Fig. 4G). Total intake over the 8 sessions showed a trend towards main effect of Sex (F_(1,55)_ = 3.067, *p* = 0.0855) and Shock (F_(1,55)_ = 0.4489, *p* = 0.5056), but no effect of Treatment nor interactions (Fig. 4H). There were no Treatment or Shock effects in Wistars of either sex on first session intake (Fig. S1M, N); However, shock VEH treated rats showed reduced total cumulative intake over the 8 sessions (*p* = 0.0488; Fig. S1O, P). In VEH treated rats, iP showed greater alcohol intake compared to Wistar rats in the return to first session post-treatment (main effect Strain, F_(1,53)_ = 22.94, *p* < 0.0001, Fig. S2H), while delta alcohol consumption between rat strains showed reduced alcohol intake in iP rats (*p* = 0.00113) but no difference in Wistars (*p* = 0.1334; Fig. S3I). Across sessions, there was an interaction between Session and rat Strain (*p* = 0.0342; Fig. S2J) in cumulative alcohol intake, but no difference between strains within any one session. Therefore, MDMA’s protective effect on shock enhanced alcohol consumption was only observed in iP rats.

### MDMA-induced changes in fear extinction are not dependent on prior alcohol exposure in iP rats

**Fig. 5:**
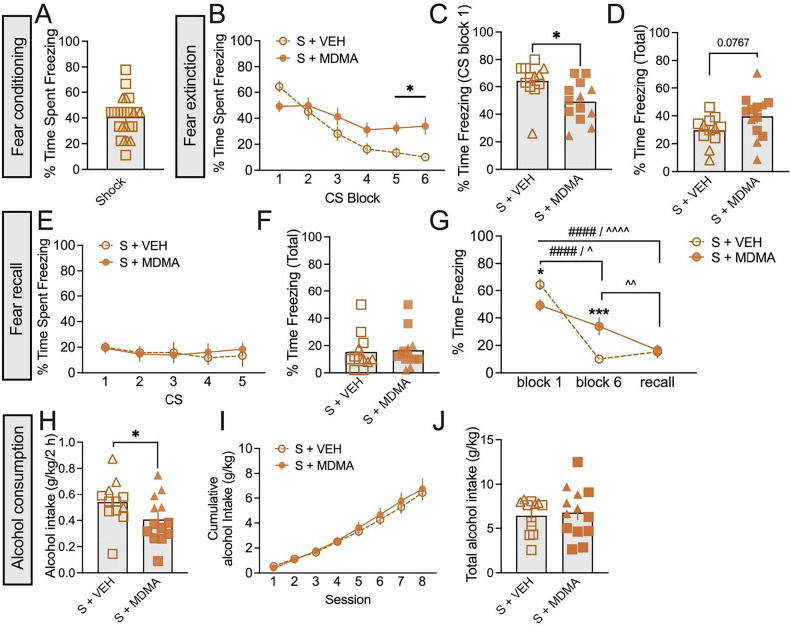
MDMA alters fear extinction in iP rats with no prior alcohol experience. **A** Percent time spent freezing during D2 fear conditioning. **B** %time spent freezing during fear extinction session by CS block. **C** In the first 10 CS during the fear extinction session, % time spent freezing in S + MDMA was significantly lower than in S + VEH. **D** Over the entire fear extinction session there was a trend towards increased freezing in S + MDMA compared to S + VEH treated rats. **E** % time spent freezing during fear recall session by CS block, (**F**) over the entire fear recall session (5 CS), **G** changes in freezing behaviour from CS block 1 to CS block 6 and recall 24 h later. **H** Male, but not female S + MDMA rats showed reduced alcohol consumption (g/kg) upon initial access compared to S + VEH rats (also see Fig. S3). **I** However, cumulative alcohol intake (g/kg), nor **J** cumulative total intake across 8 sessions did not differ between treatment groups. However, some sex differences were observed (for further analysis separated by sex see Fig. S4). Female triangle symbols, male square symbols. Data analysed by three-way ANOVA with Bonferroni *post-hoc*, *p < 0.05 ***p < 0.001. In **G**: * indicates significant between vehicle and MDMA treatment groups at discrete timepoints; # denotes significance within VEH groups across timepoints, ^ denotes significance within MDMA groups across timepoints Data represented as mean ± SEM. S shock, NS no shock, VEH vehicle.

Given our results show the effects of MDMA were specific to cohorts with prior fear conditioning, we next assessed fear conditioning and subsequent alcohol use in a cohort of alcohol-naïve iP rats to isolate the drug effects from alcohol history. No difference was observed between sexes in freezing to CS presentation on fear conditioning day 2 (unpaired t-test, *t* = 0.9649, df=23, *p* = 0.3446; Fig. 5A). Further, no main effect of sex was observed in freezing across the extinction session. Two-way ANOVA showed a main effect of CS block (F_(5,115)_ = 34.77, *p* < 0.0001), and CS block x Treatment interaction (F_(5,115)_ = 7.895, *p* < 0.0001) and a trend for Treatment (F_(1,23)_ = 3.436, *p* = 0.0767). Bonferroni *post-hoc* analysis showed higher freezing in MDMA- than VEH-treated rats by the final CS blocks (blocks 5–6: *p* = 0.0486 and *p* = 0.0054 respectively; Fig. 5B). Further, MDMA reduced freezing during the first 10 CS of fear extinction with a main effect of treatment (F_(1,21)_ = 7.375, *p* = 0.0130); a trend towards Sex (F_(1,21)_ = 3.358, *p* = 0.0811); but no interaction (Fig. 5C). *Post-hoc* analysis did not show any significance; however, analysis was separated by sex, a significant difference was observed in males (*t* = 2.454, df=14, *p* = 0.0278; Fig. S4B). No effect of Sex was observed in total % time spent freezing. With sexes combined a trend towards main effect of treatment was observed (unpaired t-test *t* = 1.854, df=23, *p* = 0.0767) with MDMA-treated animals trending higher than VEH-treated counterparts (Fig. 5D). Analysis of pre-CS baseline motion showed no significant effect of MDMA (Fig. S3C). At drug-free recall the next day, again no sex differences were observed. Two-way ANOVA showed no main effect of treatment, CS or interaction (Fig. 5E) nor were any sex differences or effect of treatment observed on total % time freezing (t = 0.2415, df=23, *p* = 0.8113, Fig. 5F). Comparing freezing across extinction block 1, block 6 and recall found a trend towards effect of Sex (F_(1,21)_ = 3.497, *p* = 0.0755), a main effect of Time (F_(1.685,35.38)_ = 58.00, *p* < 0.0001) and a Time by Treatment interaction (F_(2,42)_ = 12.21, *p* < 0.0001; Fig. 5G, Fig. S4C, D). *Post-hoc* analysis confirmed more freezing in VEH than MDMA animals during extinction block 1 (*p* = 0.0125), but conversely more freezing in MDMA than VEH animals during block 6 (*p* = 0.0002), and no difference between treatment groups at recall. Compared with block 1, both VEH and MDMA animals froze less at block 6 (VEH p < 0.0001; MDMA *p* = 0.0144) and recall (VEH *p* < 0.0001; MDMA *p* < 0.0001). In the alcohol-naïve iP cohort, MDMA-treated animals showed lower freezing at drug-free recall than during late extinction (*p* = 0.0048), despite elevated freezing late in the on-drug extinction session. This supports the interpretation that MDMA alters within-session fear expression (extinction performance) without producing lasting changes in extinction recall.

When assessing post-treatment first-access alcohol consumption, a main effect of Treatment (F_(1,21)_ = 6.447, *p* = 0.0191) and Sex (F_(1,21)_ = 23.76, *p* < 0.0001), but no interaction were observed. *Post-hoc* analysis showed that MDMA-treated males consumed significantly less alcohol (g/kg) than VEH-treated males (*p* = 0.0488), but no difference in females (*p* > 0.9999; Fig. 5H and Fig. S4E-F). Analysis of cumulative alcohol consumption across 8 sessions showed a main effect of Session *(*F_(7,147)_ = 148.9, *p* < 0.0001*)*, Sex (F_(1,21)_ = 10.82, *p* = 0.0035), Sex x Session interaction (F_(7,147)_ = 3.656, *p* = 0.0011), but no main effect of Treatment or other interactions (Fig. 5I and Fig. S4J, K). Total intake over the 8 sessions showed a main effect of Sex (F_(1, 21)_ = 5.185, *p* = 0.0334), but no effect of treatment or interaction (Fig. 5J, Fig. S4L, M). Together suggesting MDMA may produce some initial reductions alcohol consumption, but these are not enduring.

## Discussion

### Summary of key findings

In a rodent model of PTSD and alcohol use, we evaluated the potential of MDMA to modulate both extinction learning and subsequent alcohol consumption in inbred iP rats and outbred Wistar rats. Because alcohol can serve as self-medication for trauma-induced distress [4, 5] and MDMA can facilitate rodent conditioned-fear extinction [33, 34], we posited that MDMA would strengthen extinction memory and therefore reduce later alcohol consumption. Instead, MDMA altered acute fear expression in a strain- and sex-dependent manner without improving extinction recall, yet selectively blunted trauma enhanced alcohol consumption in alcohol-experienced iP rats. These results suggest more complex mechanisms underpin (and link) these behaviours than previously thought, and that MDMA’s therapeutic potential may lie less in enhancing fear extinction, and more in modulating acute affective states or cue-salience modulation during re-exposure to trauma-associated cues.

### Modelling co-morbid PTSD and alcohol consumption

Given calls for integrated PTSD-alcohol use models that capture the functional relationship between the two factors [1, 12], we first assessed whether our protocol produced a “trauma”-induced increase in alcohol consumption. Fear conditioning modestly, but consistently increased drinking in iP rats but not Wistar rats, despite robust fear learning in both, suggesting that the protocol may escalate intake only in rats with a vulnerable genetic background. This matches preclinical work showing that stress or aversive learning do not uniformly promote alcohol intake, but interact with timing, context, and susceptibility factors [13, 44, 45]. iP rats exhibit robust stress-induced alcohol seeking [46–48], and footshock-induced drinking increases are more pronounced in P rats than Wistar rats [49]. P rats also show greater anxiety- and stress-like behaviours than non-preferring (NP) rats [50], and alcohol’s anxiolysis varies by strain: high-preferring lines exhibit greater acute anxiolytic responses to alcohol than low-preferring lines [51, 52], including P vs. NP [50]. Because P-line rats were selectively bred from Wistar stock and show stronger alcohol-preference [53], alcohol-induced anxiolysis may be comparatively stronger - and thus more reinforcing - in our iP cohorts. This echoes human evidence linking familial AUD risk to greater sensitivity to alcohol’s anxiety-reducing effects [54, 55] and of shared genetic risk for PTSD and AUD [56–58]. Overall, the strain-bounded escalation we observed aligns with the self-medication hypothesis but is constrained to genetically vulnerable populations.

### MDMA’s effects on the extinction of conditioned fear

MDMA altered acute fear expression during extinction, without changing next-day recall, across both strains. It consistently reduced early-extinction freezing, suggesting an initial dampening of conditioned fear, yet learning curves diverged by strain and alcohol history. Notably, MDMA had opposite within-session effects across strains: reduced overall on-drug cue-freezing in (female) Wistar rats but increased in iP rats. Because alcohol can retroactively affect established fear memories and impair extinction [59, 60], iP rats’ higher cumulative pre-extinction alcohol may have impacted MDMA’s extinction effect. However, this alone cannot explain the MDMA-related freezing differences, as alcohol-naïve iP rats also showed heightened on-MDMA freezing. Importantly, altered freezing during the extinction session should not be conflated with impaired extinction learning. While MDMA increased overall freezing during the extinction session in iP rats, freezing did not differ between MDMA- and vehicle-treated animals at drug-free recall, indicating intact between-session extinction memory. Performance during extinction (i.e., within-session fear expression) and extinction recall (i.e., memory consolidation) can dissociate, such that elevated on-drug freezing does not necessarily reflect impaired extinction. Indeed, prior rodent work has shown that disrupting cannabinoid receptor 1 (CB1) signalling [61] or calcineurin-dependent processes [62] blunt within session extinction without abolishing between-session extinction, meaning performance-level differences do not imply or dictate differences in consolidation. Accordingly, our data support altered acute fear expression during extinction rather than impaired extinction learning per se.

Across alcohol histories, MDMA-treated iP rats showed similar extinction patterns – an initial drop in freezing followed by higher levels overall - but with cohort-specific trajectories. While most treatment differences disappeared by the final extinction block, MDMA-treated alcohol-naïve iP rats were the exception. In this cohort MDMA-treated rats froze significantly less at recall than late extinction, possibly reflecting MDMA-facilitated reconsolidation [35, 63] despite poorer within-session extinction. Further, only MDMA-treated (male) Wistar rats showed significantly increased recall freezing versus extinction block 6. VEH-treated male Wistar rats also significantly increased freezing when analysed by sex aligning with reports that male Wistar rats are more prone to fear renewal than females [64]. Interestingly, the significant early-session freezing drop was entirely female-driven in the Wistar cohort, consistent with female Wistar rats’ previously documented increased sensitivity to acute MDMA [65, 66]. Nonetheless, recall never differed between MDMA and VEH, suggesting MDMA neither impaired nor enhanced extinction.

### MDMA’s effect on later alcohol consumption

An interesting effect was observed in post-treatment alcohol consumption. In fear-conditioned alcohol-experienced iP rats (those for whom fear conditioning had escalated intake) extinction-adjunctive MDMA prevented the later footshock escalated alcohol consumption. This was more than a transient effect. In alcohol-naïve iP rats, MDMA produced only initial-session male-specific decreases, and Wistars were unaffected. Our results echo earlier reports that MDMA transiently reduced home-cage drinking in alcohol-preferring P and Fawn-Hooded rats [36], although interestingly, we observed no MDMA effect on alcohol consumption in no-shock controls who underwent identical tone-only exposure. This indicates that MDMA does not alter post-treatment alcohol consumption in the absence of fear conditioning, arguing against a trauma-independent or nonspecific pharmacological effect on drinking, at least in this procedure. This is consistent with studies demonstrating durable behavioural modulation only when MDMA is paired with trauma-cue reactivation [63].

### Behavioural interpretations

#### Strain effects

Alcohol-preferring iP rats differ from outbred Wistar rats across multiple behavioural and neurobiological domains relevant to stress responsivity, affect regulation, and alcohol motivation. iP rats exhibit high voluntary alcohol preference and elevated anxiety-like behaviour alongside altered monoaminergic signalling, dysregulated stress-axis function, and enhanced stress-induced alcohol seeking [67, 68]. In addition, iP rats also show impairments across several cognitive domains, including deficits in working memory, behavioural flexibility, and inhibitory control, which may influence performance in learning and extinction procedure independently of drug treatment. These traits are thought to reflect a polygenic vulnerability state arising from selective breeding rather than disruption of a single molecular pathway, and may be further shaped by experience-dependent epigenetic regulation of stress- and reward-related gene networks. While the present study did not include genetic, epigenetic or neurochemical analyses, these established strain characteristics provide a biological context for interpreting the selective sensitivity of iP rats to shock-associated increases in alcohol consumption. Further, mechanistically, MDMA acutely elevates synaptic monoamines (5-HT, NE, DA) and shifts neurohormonal state (via downstream oxytocin and corticosterone) [30, 69], therefore, baseline differences in monoaminergic tone could shape strain responses to MDMA or align with prior reports of opposite thermoregulatory [36] and anxiety [70] responses to MDMA.

#### Salience modulation vs. extinction enhancement

Across fear and alcohol outcomes, our data converge on a more constrained interpretation: MDMA does not reliably strengthen extinction memory in this procedure but alters fear expression during re-exposure of trauma cues in ways that may influence later alcohol consumption (within genetically- and experientially-vulnerable populations). This framing is grounded in the clinically approved use of MDMA exclusively as an adjunct to trauma-focused psychotherapy, in which drug administration is explicitly paired with cue re-exposure sessions rather than delivered in isolation. Accordingly, our interpretation of results is based off the dissociation between altered within session fear responses and unchanged fear recall, rather than direct measures of threat-safety discrimination. This interpretation aligns with human experimental findings showing MDMA impairs encoding and retrieval of emotional, but not neutral, memories [71], indicating modulation of emotional salience rather than enhanced learning per se. Further, MDMA did not improve the overall extinction of fear potentiated startle [31, 32] and reductions in physiological fear (skin-conductance) responses have been reported at the cost of poorer threat–safety discrimination [32]. These patterns are more consistent with fear-expression modulation than facilitation of extinction learning. Consistent with this, neural regulation of conditioned fear differs for acquisition vs expression [72], allowing acute changes in fear responding without altering extinction memory.

Although these findings counter traditional accounts framing MDMA as increasing tolerability of cue exposure [30], they align with clinical reports of transiently heightened in-session anxiety [73]. Importantly, heightened fear expression while on drug did not preclude downstream reductions in alcohol consumption, indicating that MDMA’s acute state effects do not map directly onto extinction outcomes or drinking behaviour. Overall, our results support emerging clinical data of broad and durable reductions in alcohol use when MDMA is delivered in a therapeutic context [19–21]. If MDMA functions as a neuromodulatory amplifier of affect regulation or cue salience - rather than as a stand-alone extinction enhancer - its capacity to influence alcohol consumption may be greatest when trauma and alcohol use are functionally linked, and transient drug-induced states can be integrated through adjunctive therapy.

### Limitations and translational implications

Several caveats constrain interpretation. Because alcohol intake was assessed under limited-access conditions and BECs were not measured, the observed changes reflect shifts in voluntary consumption rather than necessarily reaching intoxication-level exposure. Further, we tested a single MDMA dose and timing (5 mg/kg, i.p., 30 min pre-extinction); dose–response and alternative schedules may reveal conditions with differing extinction or drinking effects [76]. We did not collect core-temperature telemetry, which could help parse thermoregulatory effects previously observed in P rats [36]. Sex- and strain-specific patterns emerged that our design was inadequately powered to test, warranting follow-up. Further, while strain differences were observed, cohorts were not run in parallel due to logistical reasons, which limits direct comparisons. Finally, extinction-based procedures model only one element of trauma therapy, cue re-exposure, and cannot capture the broader relational and integrative context of MDMA-assisted psychotherapy, where prosocial and alliance-building effects may be central [30].

Within these bounds, several translational implications follow. First, MDMA’s capacity to reduce alcohol consumption after a period of abstinence may be dependent on the existence of a trauma-drinking contingency and on genetic vulnerability for high alcohol reinforcement. In clinical terms, this suggests that MDMA-adjunctive interventions may be most helpful for PTSD-AUD patients whose drinking is tightly bound to trauma-linked negative affect, rather than as a general anti-craving agent. Second, the absence of enhanced extinction in this proctocol counsels against framing MDMA as an “extinction drug”. A more accurate description may be a facilitator of affect regulation and salience recalibration during exposure-based processes - an interpretation consistent with human studies and with the therapeutic context where durable clinical gains have been reported [17, 20, 31, 32], implying psychedelic/MDMA-assisted therapy is a complex intervention where drug and psychotherapy need to be specified together [74]. Third, MDMA did not increase alcohol consumption in any group; concerns that an acutely psychoactive agent might potentiate subsequent substance use [24] were not borne out.

## Conclusions

Collectively, our data indicate that MDMA’s effects derives not from direct facilitation of extinction memory but possibly from altered cue salience during trauma retrieval, shaping a lasting modulation of affective state. This may in turn interrupt later drinking drive in vulnerable populations, while leaving fear extinction-learning processes unchanged. Translationally, these findings suggest that while MDMA-assisted psychotherapy may reduce alcohol risk in vulnerable contexts, its efficacy likely depends on therapeutic framing and individual susceptibility. More broadly, understanding MDMA as a modulator of state and salience underscores the importance of integrated treatment approaches, in which trauma-focused therapy and relapse prevention are addressed together rather than sequentially. In this light, our results not only inform mechanistic debates about how MDMA works, but also reinforce the clinical need to design interventions that target the intertwined nature of PTSD and alcohol use.

## Supplementary information

supplementary files - clean

## Data Availability

All data will be made available upon reasonable request to the corresponding author.
