# Supplementary material for: MDMA alters fear extinction, and reduces alcohol consumption in inbred alcohol preferring iP rats but not outbred Wistar rats
Source: Neuropsychopharmacology. 2026 Mar 27;51(9):1556–67. doi: 10.1038/s41386-026-02394-2 (PMC13389210; doi:10.1038/s41386-026-02394-2)
Supplement: Supplementary file 1 — supplementary files - clean [file 41386_2026_2394_MOESM1_ESM.docx]

**Huckstep, Newton et al. Supplementary materials**

**Supplementary Methods**

**Animals**

Male and female alcohol-preferring iP rats (cohort 1, n = 53, 28 female, 25 male; cohort 3 n = 25, 9 female, 16 male) originally obtained from TK Li (when at Indiana University), were bred in house at the Florey Institute, Melbourne, Australia. Male and female Wistar rats (cohort 2, n = 64, 32 female, 32 male) aged 6-7 weeks were obtained from the Animal Resource Centre, Perth, Australia. All rats were habituated for 7 days to the light/dark cycle before being individually housed in open-top cages. Rats were maintained on a reverse light cycle (lights off at 7.00 – 19.00) under temperature-controlled conditions (20 +/- 2.C, humidity ~40%). Rats had *ad libitum* access to standard chow (laboratory chow, Barastoc) and water, except where stated, with behavioural experiments conducted during the dark phase (10.00 - 14.00). All experiments were conducted in accordance with the Prevention of Cruelty to Animals Act, under the guidelines of the National Health and Medical Research Council (NHMRC) Australian Code of Practice for the Care and Use of Animals for Experimental Purposes. Approved by The Florey Animals Ethics Committee (AEC 22-013-FINMH).

**Drugs**

MDMA hydrochloride (BDG Synthesis, LOT BDG 10480.1) was dissolved in saline and injected at 5 mg/kg, 1ml/kg, i.p (Rezvani et al., 1992). Rats were habituated to injections at least 3 times prior to test.

**Fear Conditioning**

*Apparatus*

Rats underwent fear conditioning in chambers equipped with Med Associates VideoFreeze System (Med Associates, VT) enclosed in a sound attenuating box (31.8 cm x 25.4 cm x 26.7 cm) with a near Infra-red (NIR) light source. Each chamber was connected with a speaker to deliver an auditory tone and monochrome camera with a NIR pass filter to record behaviour of the rats during experimental sessions. Floor grids were connected to a shock generator (Med Associates) to deliver foot shock.

*Procedures*

*Conditioning:* On day 1 fear conditioning, rats were placed into the novel conditioning chambers and after two minutes baseline recording, the conditioned stimulus tone (CS; 5000 Hz, 80 dB) was presented for 10 seconds, co-terminating with a 1 second foot shock (unconditioned stimulus; US, 1 mA). Rats received 10 CS-US pairings [inter-trial interval (ITI; 240-480 seconds)]. No shock control rats underwent the same protocol, however, they received the tone alone. On day 2, shock rats received one CS-US (tone–shock) pairing after 230 seconds of baseline. No shock controls received the tone without shock. Bedding was changed and chambers cleaned between rats.

*Extinction:* To assess whether MDMA alters fear extinction, all rats were placed into the fear conditioning chambers 30 minutes after MDMA (5mg/kg i.p.) or saline (1 ml/kg, i.p.) administration. After a 2-minute baseline, the CS (10 s tone cue 5000 Hz, 80 dB) was presented 60 times with 10 s ITI. No shock was delivered. Bedding was changed and chambers cleaned between rats.

*Recall:* To test recall of fear behaviour without the acute effects of MDMA onboard, rats were placed back into the fear chambers 24 hours after MDMA administration and received 5 CS exposures without shock delivery (10 s tone cue 5000 Hz, 80 dB, 10 sec ITI) after a 2-minute baseline. Chambers were cleaned between each rodent.

Automated motion (total movement) and freezing responses were collected using VideoFreeze (Med Associates), set at a motion threshold >50 and freezing for at least 30 frames (1 second) and confirmed by experimenters blinded to condition (Ganella et al., 2017).

**Alcohol Drinking Procedures**

*Pre-treatment:* Initially, to habituate rats to alcohol they received continuous access for 48 hours in a two-bottle choice procedure (20% v/v ethanol bottle and water bottle), consumption and preference for alcohol were monitored at the 24- and 48-hour timepoints (cohort 1 & 2). To produce high levels of alcohol intake, rats from cohort 1 & 2 were then given voluntary access to 20% v/v ethanol in a two-bottle, intermittent modified drinking in the dark protocol (Maddern et al., 2024, Pearl et al., 2025, Walker et al., 2020). Briefly, rats completed 18 binge drinking sessions over 6 weeks, where 3 times per week (Monday, Wednesday, Friday) for 2 hours (10.00 - 12.00) rats had access to a 20% v/v ethanol bottle and water bottle. Cohort 3 did not have access to alcohol prior to fear extinction.

*Post-treatment:* To assess whether MDMA during fear extinction alters subsequent alcohol consumption, rats were given access to 20% v/v ethanol in a two-bottle choice protocol 24 hours after fear recall. Rats had continued access 3 times a week, for 2 hours, 3 hours into the dark phase (10.00 – 12.00). The total consumption in grams per kilogram (g/kg) was calculated using the volume consumed in millilitres (mL), multiplied by the density of 20% v/v ethanol, and divided by the rat weight = ((mL alcohol consumed x percentage of alcohol x density of 20% v/v ethanol)/ (weight of rat (g)/1000)).

**Statistical Analysis**

All statistical analysis was performed through GraphPad Prism 10 (GraphPad Software Inc, LLC), with significance set at p < 0.05. All data figures are represented as mean ± SEM. Two- or three-way ANOVAs were used for all analysis (see Table 1 for full details) depending upon the number of independent variables. If values were missing (e.g. due to bottle spillage) a mixed effect analysis was undertaken. Where no effect of sex was observed, data were reanalysed using two-way ANOVA (or unpaired t-test, where appropriate) to pool sexes together. Where sex differences were observed, analysis was subsequently undertaken with sexes separated (Fig. S1 and S4). If significance (p<0.05) or a trend (p<0.1) was observed Bonferroni *post-hoc* analyses were performed to adjust for repeated measures. One iP rat (from cohort 1) was excluded from analysis of post-treatment alcohol consumption due to excessive spillage.

**References:**

Ganella, D. E., Barendse, M. E., Kim, J. H., & Whittle, S. (2017). Prefrontal-amygdala connectivity and state anxiety during fear extinction recall in adolescents. *Frontiers in human neuroscience*, *11*, 587.

Maddern, X. J., Letherby, B., Ch’ng, S. S., Pearl, A., Gogos, A., Lawrence, A. J., & Walker, L. C. (2024). Cocaine and amphetamine regulated transcript (CART) mediates sex differences in binge drinking through central taste circuits. *Neuropsychopharmacology*, *49*(3), 541-550.

Pearl, A. J., Maddern, X. J., Pinares-Garcia, P., Ursich, L. T., Anversa, R. G., Shesham, A., ... & Walker, L. C. (2025). Midbrain ghrelin receptor signalling regulates binge drinking in a sex specific manner. *Nature Communications*, *16*(1), 2568.

Rezvani, A. H., Garges, P. L., Miller, D. B., & Gordon, C. J. (1992). Attenuation of alcohol consumption by MDMA (ecstasy) in two strains of alcohol-preferring rats. *Pharmacology Biochemistry and Behavior*, *43*(1), 103-110.

Walker, L. C., Berizzi, A. E., Chen, N. A., Rueda, P., Perreau, V. M., Huckstep, K., ... & Lawrence, A. J. (2020). Acetylcholine muscarinic M4 receptors as a therapeutic target for alcohol use disorder: converging evidence from humans and rodents. *Biological Psychiatry*, *88*(12), 898-909.

**Supplementary Figures
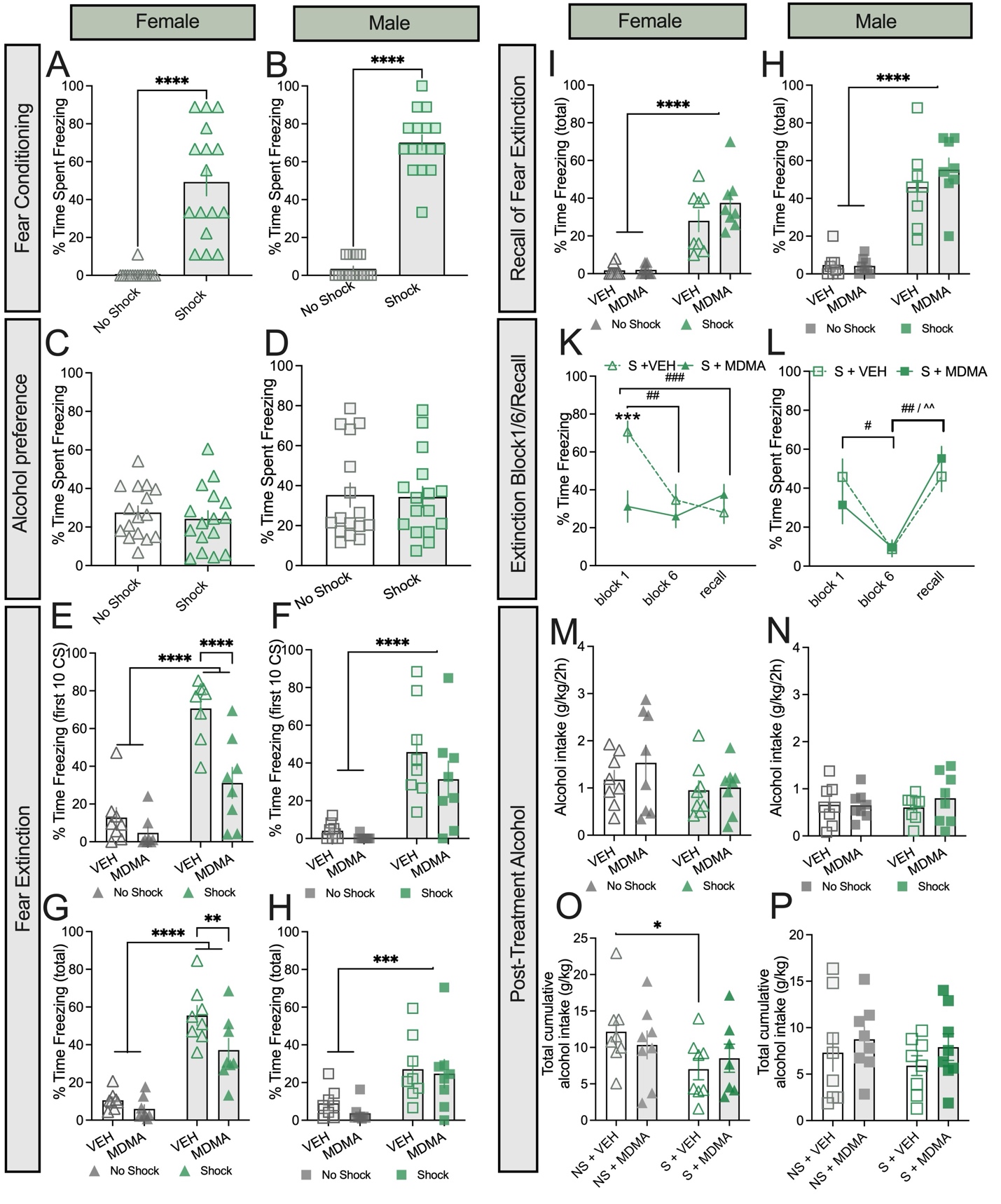
**

**Supp. Figure S1. Sex differences in Wistar Rats.** A sex difference in freezing behaviour during conditioning was observed in Wistar rats, and further analysis separated by sex showed **(A)** female rats exposed to footshock increased their freezing on day 2 conditioning (t=6.682, df=30, p<0.0001) and **(B)** Male rats exposed to foot shock also increased their freezing on day 2 conditioning (t=15.74, df=30, p<0.0001). A trend toward Sex was observed in alcohol preference, however, sex separated unpaired t-test showed no effect of Shock in either **(C)** female (t=0.6177, df=30, p=0.5415), or **(D)** male Wistar rats (t=0.1219, df=30, p=0.9038). Sex differences in fear extinction were also observed, two-way ANOVA separated by sex revealed **(E)** female rats with a history of footshock showed reduced freezing during the first 10 CS following in rats after MDMA administration compared to VEH treated rats (main effect treatment F_(1, 28)_ = 16.61, p=0.0003; main effect shock F_(1, 28)_ = 52.22, p<0.0001; and shock x treatment interaction F_(1, 28)_ = 7.223, p=0.0120). Bonferroni *post-hoc* showed a significant difference between shock VEH and shock MDMA (p<0.0001). **(F)** While in male rats, a main effect of shock (F_(1, 28)_ = 29.47, p<0.0001), but no effect of treatment (F_(1, 28)_ = 1.823, p=0.1877), nor interaction (F_(1, 28)_ = 0.6454, p=0.4285) was observed. A sex difference in total freezing time during fear extinction was also observed. Further analysis separating sex showed **(G)** female rats with a history of foot shock showed reduced freezing during the total extinction session after MDMA administration compared to VEH treated rats (two-way ANOVA, main effect treatment F_(1, 28)_ = 7.149, p=0.0124; main effect shock F_(1, 28)_ = 78.96, p<0.0001; but no shock x treatment interaction F_(1, 28)_ = 2.557, p=0.1210). Bonferroni *post hoc* showed a significant difference between shock VEH and shock MDMA (p=0.0053). **(H)** While in male rats, a main effect of shock (F_(1, 28)_ = 14.54, p=0.0007), but no effect of treatment (F_(1, 28)_ = 0.5571, p=0.4616), nor interaction (F_(1, 28)_ = 0.08419, p=0.7738) was observed. A sex difference in recall freezing was also observed in Wistar rats, however both **(I)** female and **(J)** male rats showed no main effect of treatment (female, F_(1, 28)_ = 1.501, p=0.2307; male, F_(1, 28)_ = 0.7328, p=0.3992), only a main effect of shock (female, F_(1, 28)_ = 60.23, p<0.0001; male, F_(1, 28)_ = 81.46, p<0.0001). A significant sex x time interaction was observed when analysing freezing across extinction block 1 – block 6 – recall, and further analysis separating sex revealed **(K)** reduced freezing across time in female VEH but not MDMA rats (two-way ANOVA, main effect treatment F_(1, 14)_ = 5.235, p=0.0382; main effect time F_(1.869, 26.16)_ = 5.998, p=0.0082; shock x treatment interaction F_(1.869, 26.16)_ = 7.268, p=0.0036). Bonferroni *post-hoc* analysis showed a difference between Shock VEH and Shock MDMA in block 1 (p = 0.0001), but not block 6 (p = 0.3634) or recall (p = 0.3136). Further Shock VEH rats showed differences between block 1 and block 6 (p=0.0016) and block 1 and recall (p=0.002), but not block 6 and recall (p>0.9999). Shock MDMA rats showed no significant difference between any blocks (p’s>0.6000). **(L)** In contrast males showed only a main effect of time (F_(1.905, 26.67)_ = 22.29, p<0.0001) but not treatment nor interaction. Bonferroni *post-hoc* analysis showed Shock VEH rats differed between block 1 and block 6 (p=0.0144) and block 6 and recall (p = 0.0089), but not block 1 and recall (p>0.9999). Shock MDMA rats differed between block 6 and recall (p=0.0033), but not block 1 and block 6 (p=0.2498) and a trend between block 1 and recall (p>0.0568). Similarly a sex difference in alcohol consumption was also observed, however once separated by sex no significant differences were observed in **(M)** female (two-way ANOVA no effect of treatment F_(1, 28)_ = 0.6759, p=0.4179; shock F_(1, 28)_ = 2.199, p=0.1493; or interaction F_(1, 28)_ = 0.3535, p=0.5569) or **(N)** male (two-way ANOVA, no main effect of treatment F_(1, 28)_ = 0.4361, p=0.5144; shock F_(1, 28)_ = 0.1152, p=0.7368; or interaction F_(1, 28)_ = 0.5466, p=0.4659) Wistar rats when analysed separately by sex. Similarly for total cumulative intake, a trend towards main effect was observed, when separated by sex **(O)** female Wistar rats showed no main effect of Treatment (F_(1, 27)_ = 0.007835, p=0.9301); no interaction (F_(1, 27)_ = 0.8526, p=0.3640); but a trend towards effect of Shock F_(1, 27)_ = 3.779, p=0.0624. *Post-hoc* analysis showed Shock + VEH rats consumed less alcohol than no shock controls (p=0.0488). **(P)** Male Wistar rats showed no main effect of Shock (F_(1, 28)_ = 0.5591 p=0.4608), Treatment (F_(1, 28)_ = 1.316, p=0.2611), or interaction (F_(1, 28)_= 0.03334, p=0.8564). Female triangle symbols, male square symbols. Data analysed by students t-test or two-way ANOVA with Bonferroni *post-hoc*, ****p<0.0001, ***p<0.001, **p<0.01, *p<0.05. In (I) and (J): * denotes significant difference between treatment groups at a discrete timepoint, # denotes significant effect between timepoints within the VEH group and ^ denotes significance between timepoints within the MDMA group. Data represented as mean±SEM. Abbreviations: NS = no shock, S = shock


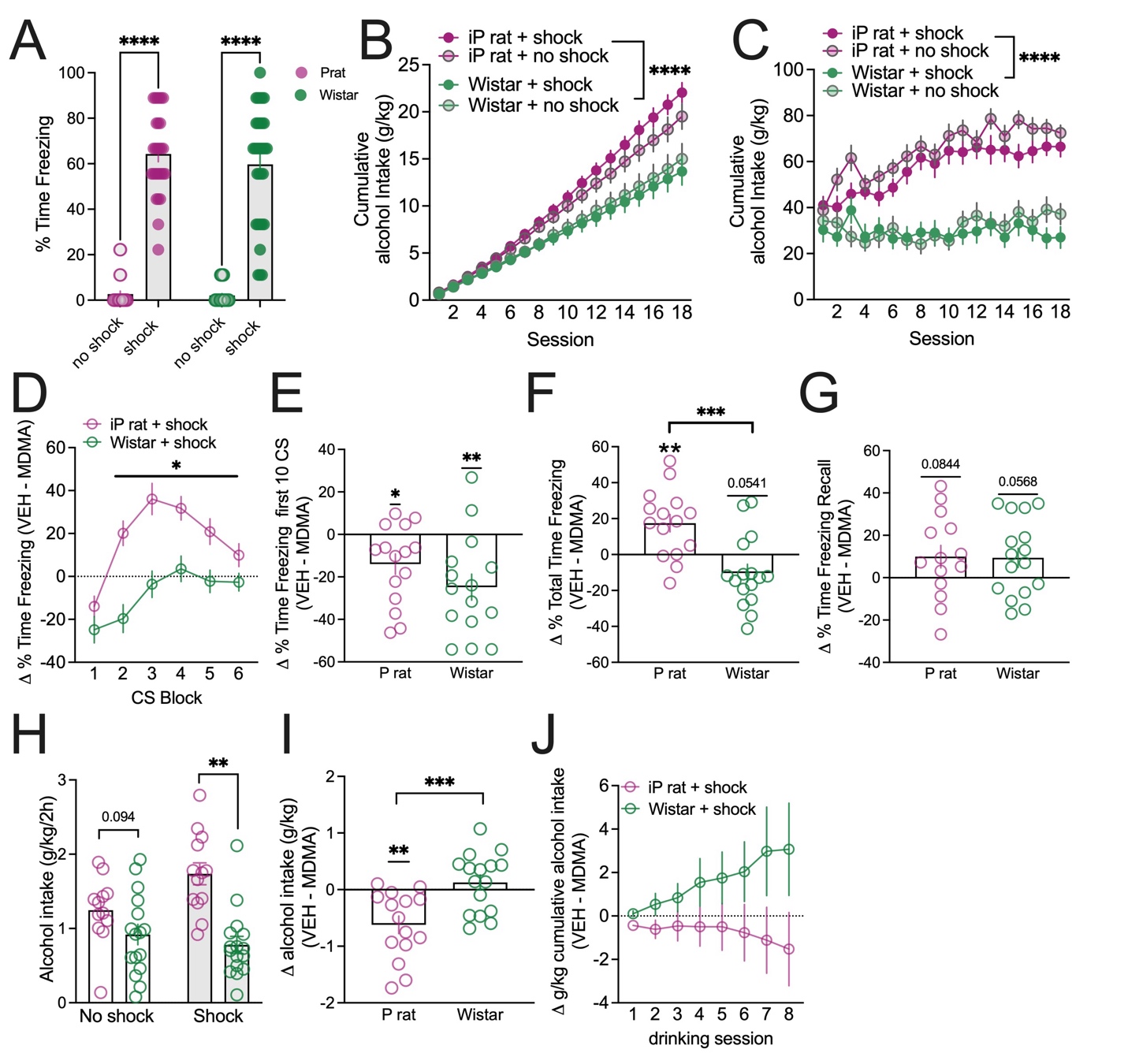


**Supp. Figure S2. Comparison of iP and Wistar rats.** **(A)** Both iP and Wistar rats showed freezing response to the tone on day 2 conditioning, and this did not differ between the strains (two-way ANOVA, main effect shock F_(1, 113)_ = 383.4, p<0.0001; no effect strain F_(1, 113)_ = 0.7141, p=0.3999; no interaction F_(1, 113)_ = 0.4271, P=0.5148). **(B)** iP rats had significantly higher cumulative alcohol intake (g/kg) than Wistar rats (three-way ANOVA, main effect strain, F_(1, 63)_ = 16.73, p=0.0001; main effect of time F_(17, 1071)_ = 505.4, p<0.0001; strain x time interaction F_(17, 851)_ = 23.20, p<0.0001. **(C)** iP rats also showed increased alcohol preference compared to Wistar rats (three-way ANOVA, main effect: strain F_(1, 62)_ = 113.9, p<0.0001; session F_(17, 1054)_ = 8.912, p<0.0001 and time x strain interaction, F_(17, 855)_ = 6.685, p<0.0001). **(D)** Delta percent time spent freezing during fear extinction (VEH – MDMA), by CS block showed a significant difference in the effect of MDMA on freezing behaviour due to extinction between rat strains (three-way ANOVA, main effect strain F_(1, 63)_ = 16.73, p= 0.0001; main effect time F_(17, 1071)_ = 505.4, p<0.0001; time x strain interaction F_(17, 851)_ = 23.20, p<0.0001). **(E)** In first 10 CS of fear extinction, both iP and Wistar rats showed a decreased delta % time freezing (one sample Wilcoxon test, p=0.0112 and p=0.0012 respectively), however, no difference was observed between iP and Wistar rats (unpaired t-test, p=0.1729). **(F)** Over the entire session (60 CS), iP rats showed an increased delta % time freezing (one sample Wilcoxon test, p=0.0029), while Wistar rats showed a trend towards decreased delta % time freezing (one sample Wilcoxon test, p=0.0541), and a significant difference between iP rats and Wistar rats (unpaired t-test, p=0.0004). **(G)** Both iP and Wistar rats showed trends toward increased delta % time freezing during recall (one sample Wilcoxon test, p=0.0844 and p=0.0568 respectively), but no difference between rat strains (unpaired t-test, p=0.9353). **(H)** VEH treated iP rats show greater alcohol intake compared to Wistars on return to alcohol consumption (Two-way ANOVA, main effect: strain F_(1, 53)_= 22.94, p < 0.0001 and strain x shock interaction F_(1, 53)_ = 5.529, p=0.0225, but no main effect of shock F_(1, 53)_= 1.718, p=0.1956). *Post-hoc* analysis showed a trend towards increase in no shock (iP vs Wistar, p=0.0940) and significant difference in shock groups (iP vs. Wistar, p<0.0001). **(I)** iP rats showed a reduction in delta alcohol intake after MDMA administration (one sample Wilcoxon test, p=0.00113), however Wistar rats showed no difference (p=0.3470), and a significant difference was observed between the strains (unpaired t-test, p=0.0008). **(J)** Delta cumulative alcohol intake (g/kg) following fear conditioning and extinction showed an interaction between session and rat strain (two-way ANOVA, F_(7, 189)_ = 2.222, p=0.0342; but no effect of strain F_(1, 27)_ = 2.395, p=0.1334 or time F_(7, 189)_ = 0.6807, p=0.6883). Bonferroni post hoc analysis showed no significant difference for any individual session (p’s>0.05). Data analysed by students t-test or two-way ANOVA with Bonferroni *post-hoc*, ****p<0.0001, ***p<0.001, **p<0.01, *p<0.05. In (I) and (J): * denotes significant difference between treatment groups at a discrete timepoint, # denotes significant effect between timepoints within the VEH group and ^ denotes significance between timepoints within the MDMA group. Data represented as mean±SEM. Abbreviations: NS = no shock, S = shock

**
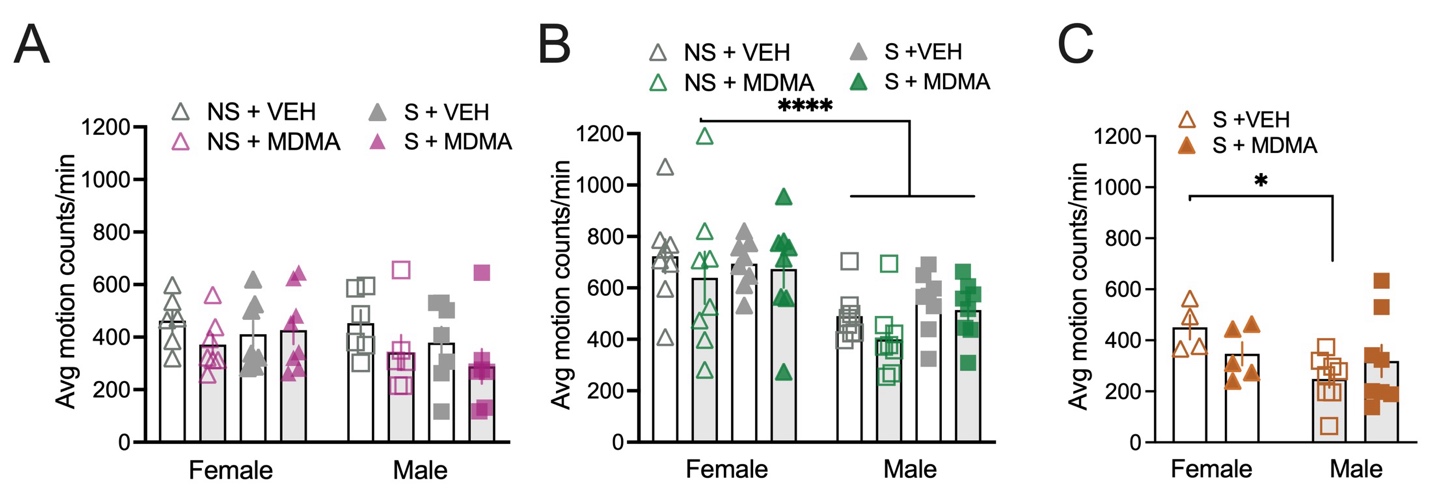
**

**Supp Figure S3. MDMA does not alter motion, but baseline sex differences exist.** When assessing average motion during baseline of the fear extinction protocol (A) in iP rats with prior alcohol exposure three-way ANOVA showed a trend toward main effect of Sex (F_(1, 46)_ = 3.244, p=0.0782) but no effect of MDMA (F_(1, 46)_ = 1.820, p=0.1839), Shock (F_(1, 46)_ = 0.6390, p=0.4282), nor interactions (Sex x MDMA, F_(1, 46)_= 0.6701, p = 0.4172; Sex x Shock, F_(1, 46)_ = 0.6763, p=0.4151; MDMA x Shock F_(1, 46)_ = 0.7174, p=0.4014; MDMA x Sex x Shock, F_(1, 46)_ = 0.3054, p=0.5832). *Post hoc* analysis showed no difference between sexes of treatment groups (p’s>0.05). **(B)** In Wistar rats with prior alcohol exposure a main effect of sex was observed (F_(1, 56)_ = 21.85, p<0.0001), but no main effect of MDMA (F_(1, 56)_ = 1.088, p=0.3015) or Shock (F_(1, 56)_ = 1.833, p=0.1812) nor interactions (Sex x MDMA, F_(1, 46)_= 0.6701, p=0.4172; sex x shock, F_(1, 46)_ = 0.6763, p=0.4151; MDMA x shock F_(1, 46)_ = 0.7174, p=0.4014; MDMA x Sex x Shock, F_(1, 46)_ = 0.3054, p=0.5832). *Post hoc* analysis showed trends towards sex differences between vehicle treated rats (No shock male vs. female p = 0.0823 and Shock female vs. male p = 0.0676). **(C)** In alcohol naïve iP rats two-way ANOVA showed a main effect of sex (F_(1, 21)_ = 4.481, p = 0.0464), but no main effect of MDMA (F_(1, 21)_ = 0.09278, p = 0.7637), nor interaction (F_(1, 21)_ = 2.563, p = 0.1243). *Post hoc* analysis revealed the difference between sexes significant in VEH (male vs. female p = 0.0378), but not MDMA-treated rats (male vs. female p = 0.9152). *Female triangle symbols, male square symbols. Data analysed by students t-test or two-way ANOVA with Bonferroni post-hoc, ****p<0.0001, ***p<0.001, **p<0.01, *p<0.05. Data represented as mean ± SEM. Abbreviations: NS = no shock, S = shock*


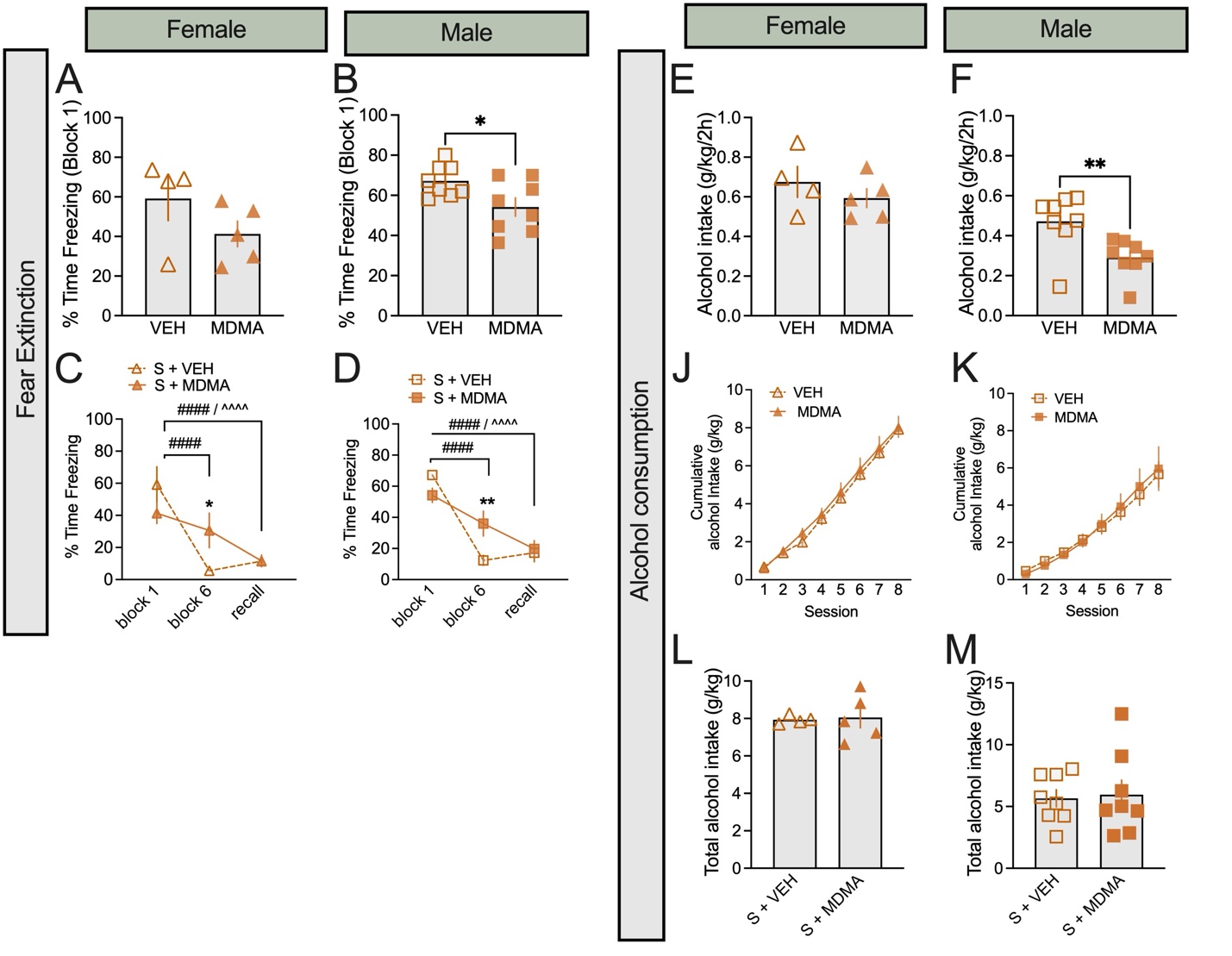


**Supp. Figure S4. Sex differences in alcohol-naïve iP rats**. In the alcohol-naïve iP cohort, analysis of freezing to the fist 10CS of extinction showed a trend towards a sex effect. Further sex-separated analyses (students t test) revealed **(A)** no significant difference between MDMA and VEH groups in females (t=1.468, df=7, p=0.1855) but **(B)** significantly less freezing in MDMA animals than VEH in males (t=2.454, df=14, p=0.0278). Further analysis showed a trend towards main effect of sex on within and between session extinction. Separation of sex sexes in **(C)** Female and **(D)** male iP rats showed effects on freezing across and within extinction sessions. Specifically, in females a main effect of Block (F_(2, 14) =_ 26.45, P<0.0001), and block x treatment interaction (F_(2, 14)_= 7.183, P=0.0071) with post hoc showing a trend towards effect of treatment at block 1 (p=0.0982) and significance at Block 2 (p=0.0243). VEH rats showed within session extinction (Block 1 vs. Block 6, p>0.0001), and between session changes from Block 1 to recall (p=0.0002), but no difference between block 6 and recall (p>0.9999). MDMA rats did not show within session extinction (Block 1 vs. Block 6, p=0.5332), but a significant difference between Block 1 and recall (p=0.0047) and a trend toward Block 6 to recall (p=0.0762). In males, a main effect of Block (F_(2, 28)_ = 40.15, p<0.0001), and block x treatment interaction (F_(2, 28)_ = 6.497, P=0.0048) with post hoc showing a trend towards effect of treatment at block 1 (p=0.0849) and significance at Block 2 (p=0.0027). VEH rats showed within session extinction (Block 1 vs. Block 6, p<0.0001), and between session changes from Block 1 to recall (p<0.0001), but no difference between block 6 and recall (p>0.9999). male MDMA treated rats showed a trend towards within session extinction (Block 1 vs. Block 6, p=0.0542), a significant difference between Block 1 and recall (p=0.0002) and a trend toward Block 6 to recall (p=0.0977). There was also a sex difference in first-access alcohol consumption. **(E)** MDMA had no significant effect on alcohol intake (g/kg) in female rats (t=0.9477, df=7, p=0.3748) but **(F)** MDMA significantly reduced alcohol consumption in males (t=2.989, df=14, p=0.0098). Cumulative alcohol consumption across 8 sessions also showed a sex difference, however subsequent analysis within each sex revealed no treatment group differences in either **(G)** females (Two-way RM ANOVA treatment F_(1, 7)_ = 0.2750, p=0.6162; session F_(7, 49)_ = 284.6, p<0.0001; interaction F_(7, 49)_ = 0.3006, p=0.9502) or **(H)** males (treatment F_(1, 14)_ = 0.01303, p=0.9107; session F_(7, 98)_ = 54.86, p<0.0001; interaction F_(7, 98)_ = 0.2688, p=0.9646). Further while sex differences were seen in total alcohol intake sex separated sex show now specific treatment differences in **(I)** Female (t=0.1842, df=7), or **(J)** male (t=0.2197, df=14) rats post treatment.


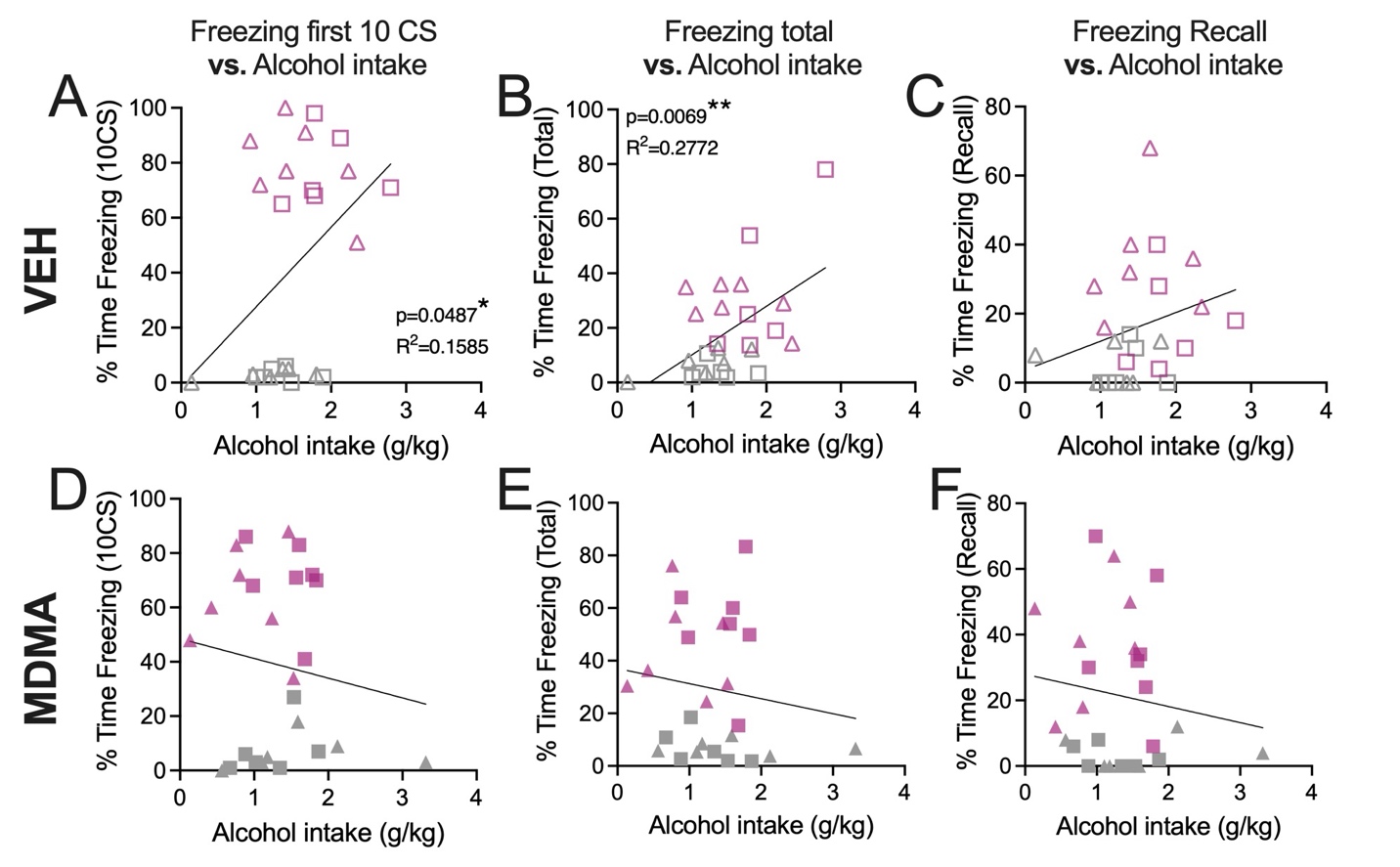


**Supp. Figure S5. Correlational analysis:** We performed correlational analyses between freezing measures and subsequent post treatment alcohol intake. While a correlation was apparent when shock (purple) and no-shock animals (grey) were combined within VEH treated iP rats for **(A)** % time freezing during first 10CS vs. alcohol intake, p=0.0487, R2 =0.1585; and **(B)** total % time freezing vs. alcohol intake, p=0.0069, R2=0.2772, **(C)** whereas freezing during recall did not correlate (p=0.2009; R2=0.07009). This relationship was lost when analyses were stratified by shock history (and was absent in other rat groups). In VEH treated iP rats fear measures appear to be primarily driven by shock status, and while increased freezing during extinction correlates with greater alcohol consumption this is not mechanistically coupled at the individual level, suggesting MDMA does not simply reduce drinking by weakening fear responding.

**Supplementary Table S1. Statistical Analyses.** This table lists all statistical tests performed for analyses presented in the main figures. “Relevant Figure/Panel” refers to the figure and panel in which each result is visualized. “Test for Sex Effect (p)” indicates the statistical test used when sex was included as a factor and its corresponding p-value. Where no trend towards, or significant sex effect was observed (p > 0.10), the subsequent reduced model used for final interpretation is listed under “Analysis Excluding Sex Factor (if appropriate).” “Details of Main Effects and Interactions” reports the F-values, degrees of freedom, and p-values for all main effects and interactions from the relevant analysis. *Significant effects (p < 0.05) are italicized within the table.*

| **Relevant Figure/Panel** | **Test for Sex Effect**  **(p)** | **Analysis Excluding Sex Factor**  **(if appropriate)** | **Details of Main Effects**  **and Interactions** |
| --- | --- | --- | --- |
| **Figure 2** | | | |
| Fig. 2B  iP fear conditioning | Two-way ANOVA  (Sex p=0.3147) | Unpaired t-test | *t=16.17, df=51, p<0.0001* |
| Fig. 2C  Wistar Fear conditioning | Two-way ANOVA  *(Sex p=0.0068)* |  | *Sex = F (1, 60) = 7.868, p=0.0068*  *Shock = F (1, 60) = 187.5, p<0.0001*  *Sex x Shock = F (1, 60) = 4.601, p=0.0360* |
| Fig 2D  iP cumulative alcohol intake | Three-way mixed effects analysis  (Sex p=0.8801) | Two-way mixed effects analysis | *Time = F (17, 863) = 522.7, p<0.0001*  Shock = F (1, 51) = 1.390 p=0.2440  *Time x Shock = F (17, 863) = 2.765, p=0.0002* |
| Fig 2E  iP alcohol preference | Three-way mixed effects analysis  (Sex p=0.4214) | Two-way mixed effects analysis | *Time = F (17, 867) = 12.21*  *p<0.0001*  *Shock = F (1, 51) = 5.045*  *p=0.0291*  Time x Shock =  F (17, 867) = 0.6440  p=0.8580 |
| Fig. 2F  iP average alcohol preference | Two-way ANOVA  (Sex p=0.2969) | Unpaired t-test | *t=2.242, df=51, p=0.0294* |
| Fig. 2G  Wistar cumulative alcohol intake | Three-way ANOVA  (Sex p=0.7323) | Two-way ANOVA | *Time = F (17, 1054) = 141.2, p<0.0001*  Shock = F (1, 62) = 0.06373, p=0.8015  Time x Shock = F (17, 1054) = 0.2141, p=0.9997 |
| Fig. 2H  Wistar alcohol preference | Three-way mixed effects analysis  (Sex p=0.0653) |  | Sex = F (1, 60) = 3.526, p=0.0653  Shock = F (1, 60) = 0.1973, p=0.6585  Session = F (17, 1019) = 1.369, p=0.1434  Sex x Shock =  F (1, 60) = 0.05929, p=0.8085  Sex x Session =  F (17, 1019) = 1.398, p=0.1289  Shock x Session =  F (17, 1019) = 1.413, p=0.1217  Sex x Shock x Session =  F (17, 1019) = 0.5959, p=0.8970 |
| Fig. 2I  Wistar average alcohol preference | Two-way ANOVA  (Sex p=0.0654) |  | *Sex =* F (1, 60) = 3.522, p=0.0654  Shock = F (1, 60) = 0.1981, p=0.6579  Time x Shock = F (1, 60) = 0.05885  p=0.8091 |
| **Figure 3** | | | |
| Fig 3A  iP fear extinction time course |  | Three-way ANOVA | *CS block = F (5, 245) = 33.85, p<0.0001*  *Shock = F (1, 49) = 79.57, p<0.0001*  *Tx = F (1, 49) = 6.315, p=0.0153*  *CS x Shock = F (5, 245) = 42.84, p<0.0001*  *CS x Tx = F (5, 245) = 6.833, p<0.0001*  *Shock x Tx = F (1, 49) = 4.328, p=0.0427*  *CS x Shock x Tx = F (5, 245) = 9.126, p<0.0001* |
| Fig 3B  iP extinction freezing first 10 CS | Three-way ANOVA  (Sex p=0.4930) | Two-way ANOVA | Tx = F (1, 49) = 1.919, p=0.1722  *Shock = F (1, 49) = 357.3, p<0.0001*  *Tx x Shock = F (1, 49) = 6.693, p=0.0127* |
| Fig 3C  iP extinction total time freezing | Three-way ANOVA  (Sex p=0.4454) | Two-way ANOVA | *Tx = F (1, 49) = 6.315, p=0.0153*  *Shock = F (1, 49) = 79.57, p<0.0001*  *Tx x Shock = F (1, 49) = 4.328, p=0.0427* |
| Fig 3D  Wistar extinction time course |  | Three-way ANOVA | *CS block = F (5, 300) = 8.844, p<0.0001*  *Shock = F (1, 60) = 57.41, p<0.0001*  *Tx = F (1, 60) = 4.038, p=0.0490*  *CS x Shock = F (5, 300) = 33.59, p<0.0001*  *CS x Tx = F (5, 300) = 4.265, p=0.0009*  Shock x Tx = F (1, 60) = 0.4975, p=0.4833  *CS x Shock x Tx = F (5, 300) = 5.324, p<0.0001* |
| Fig 3E  Wistar extinction freezing first 10 CS | Three-way ANOVA  *(Sex p=0.0387)* |  | *Sex = F (1, 56) = 4.485, p=0.0387*  *Shock = F (1, 56) = 78.15, p<0.0001*  *Tx = F (1, 56) = 13.66, p=0.0005*  Sex x Shock =F (1, 56) = 0.4321, p=0.5136  Sex x Tx = F (1, 56) = 2.757, p=0.1024  *Shock x Tx = F (1, 56) = 5.625, p=0.0212*  Sex x Shock x Tx = F (1, 56) = 1.347, p=0.2507 |
| Fig 3F  Wistar extinction total time freezing | Three-way ANOVA  *(Sex p=0.0015)* |  | *Sex = F (1, 56) = 11.19, p=0.0015*  *Shock = F (1, 56) = 74.39, p<0.0001*  *Tx = F (1, 56) = 5.232, p=0.0260*  *Sex x Shock = F (1, 56) = 7.687, p=0.0075*  Sex x Tx = F (1, 56) = 1.304, p=0.2583  Shock x Tx = F (1, 56) = 0.6446, p=0.4254  Sex x Shock x Tx = F (1, 56) = 1.558, p=0.2172 |
| Fig 3G  iP recall time course |  | Three-way ANOVA | *CS block = F (4, 196) = 3.163, p=0.0151*  *Shock = F (1, 49) = 56.98,*  *p<0.0001*  Tx = F (1, 49) = 1.465, p=0.2320  CS x Shock = *F (4, 196) = 6.578, p<0.0001*  CS x Tx = F (4, 196) = 0.8850, p=0.4739  Shock x Tx = F (1, 49) = 2.506, p=0.1198  CS x Shock x Tx = F (4, 196) = 0.8336, p=0.5053 |
| Fig 3H  iP recall total time freezing | Three-way ANOVA  (Sex p=0.1636) | Two-way ANOVA | Tx = F (1, 49) = 1.465, p=0.2320  *Shock = F (1, 49) = 56.98, p<0.0001*  Tx x Shock = F (1, 49) = 2.506, p=0.1198 |
| Fig 3I  iP within and between session extinction | Three-way ANOVA  (Sex p=0.4916) | Two-way ANOVA | *Time = F (1.738, 45.18) = 55.16, p<0.0001*  Tx = F (1, 26) = 0.1956, p=0.6620  *Time x Tx = F (1.738, 45.18) = 3.679, p=0.0388* |
| Fig 3J  Wistar recall time course |  | Three-way ANOVA | *CS block = F (4, 240) = 5.604, p=0.0003*  *Shock = F (1, 60) = 118.5, p<0.0001*  Tx = F (1, 60) = 1.710, p=0.1960  *CS x Shock = F (4, 240) = 7.890, p<0.0001*  CS x Tx = F (4, 240) = 1.472, p=0.2114  Shock x Tx = F (1, 60) = 1.804, p=0.1843  CS x Shock x Tx =F (4, 240) = 1.412, p=0.2306 |
| Fig 3K  Wistar recall total time freezing | Three-way ANOVA  *(Sex p=0.0025)* |  | *Sex = F (1, 56) = 10.02, p=0.0025*  *Shock = F (1, 56) = 141.3, p<0.0001*  Tx = F (1, 56) = 2.040  p=0.1588  *Sex x Shock = F (1, 56) = 5.544, p=0.0221*  Sex x Tx =  F (1, 56) = 0.005960, p=0.9387  Shock x Tx =  F (1, 56) = 2.152  p=0.1480  Sex x Shock x Tx =  F (1, 56) = 0.001490,  p=0.9693 |
| Fig 3L  Wistar within and between session extinction | Three-way ANOVA  (Sex p=0.248) | Two-way ANOVA | *Time = F (1.869, 56.08) = 13.75, p<0.0001*  Tx = F (1, 30) = 2.526, p=0.1224  *Time x Tx = F (1.869, 56.08) = 6.245, p=0.0043* |
| **Figure 4** | | | |
| Fig. 4A  iP first session alcohol intake post treatment | Three-way ANOVA  (Sex p=0.1822) | Two-way ANOVA | Tx = F (1, 48) = 1.727, p=0.1951  Shock = F (1, 48) = 0.9936, p=0.3239  *Tx x Shock = F (1, 48) = 4.120, p=0.0480* |
| Fig. 4B  iP first session preference | Three-way ANOVA  (Sex p=0.1490) | Two-way ANOVA | Tx = F (1, 48) = 3.498,  p=0.0676  Shock = F (1, 48) = 2.635, p=0.1111  *Tx x Shock =*  *F (1, 48) = 4.665*  *p=0.0358* |
| Fig. 4C  iP cumulative intake post treatment |  | Three-way ANOVA | *Time = F (7, 336) = 714.8, p<0.0001*  *Shock = F (1, 48) = 7.325, p=0.0094*  Tx = F (1, 48) = 1.192, p=0.2803  *Time x Shock = F (7, 336) = 6.362, p<0.0001*  Time x Tx = F (7, 336) = 1.002, p=0.4299  Shock x Tx = F (1, 48) = 2.615, p=0.1124  Time x Shock x Tx = F (7, 336) = 2.028, p=0.0510 |
| Fig. 4D  iP total cumulative alcohol intake post treatment | Three-way ANOVA  (Sex p=0.1150) | Two-way ANOVA | Tx = F (1, 48) = 1.663, p=0.2034  *Shock = F (1, 48) = 7.463, p=0.0088*  *Tx x Shock =* F (1, 48) = 3.411*, p*=0.0709 |
| Fig. 4E  Wistar first session alcohol intake post treatment | Three-way ANOVA  *(Sex p=0.0012)* |  | *Sex = F (1, 56) = 11.59, p=0.0012*  Shock = F (1, 56) = 1.281, p=0.2625  Tx = F (1, 56) = 1.080, p=0.3031  Sex x Shock = F (1, 56) = 2.135, p=0.1495  Sex x Tx = F (1, 56) = 0.1588, p=0.6918  Shock x Tx = F (1, 56) = 0.02598, p=0.8725  Sex x Shock x Tx = F (1, 56) = 0.7719, p=0.3834 |
| Fig. 4F  Wistar first session preference | Three-way ANOVA  *(Sex p=0.0144)* |  | *Sex =* F (1, 56) = 6.385*, p=0.0144*  Shock = F (1, 56) = 1.946e-005, p=0.9965  Tx = F (1, 56) = 2.591, p=0.1131  Sex x Shock = F (1, 56) = 0.8674, p=0.3557  *Sex x Tx = F (1, 56) = 6.707, p=0.0122*  Shock x Tx = F (1, 56) = 0.002066, p=0.9639  Sex x Shock x Tx =  F (1, 56) = 0.5630, p=0.4562 |
| Fig. 4G  Wistar cumulative alcohol intake post treatment |  | Three-way ANOVA | Time = F (7, 413) = 123.1, p<0.0001  Shock = F (1, 59) = 0.5211, p=0.4732  Tx = F (1, 59) = 1.532, p=0.2207  Time x Shock = F (7, 413) = 0.4492, p=0.8706  Time x Tx = F (7, 413) = 1.073, p=0.3801  Shock x Tx =F (1, 59) = 0.7936, p=0.3766  Time x Shock x Tx = F (7, 413) = 1.447, p=0.1846 |
| Fig. 4H  Wistar total cumulative alcohol intake post treatment | Three-way ANOVA  (Sex p=0.0855) |  | Sex =  F (1, 55) = 3.067, p=0.0855  Shock = F (1, 55) = 3.906, p=0.0531  Tx = F (1, 55) = 0.4489, p=0.5056  Sex x Shock =  F (1, 55) = 1.029, p=0.3149  Sex x Tx = F (1, 55) = 0.6500, p=0.4236  Shock x Tx =  F (1, 55) = 0.6851, p=0.4114  Sex x Shock x Tx = F (1, 55) = 0.3514, p=0.5558 |
| **Figure 5** | | | |
| Fig 5A  iP fear conditioning |  | Unpaired t-test | t=0.9649, df=23, p=0.3446 |
| Fig 5B  iP fear extinction time course | Three-way ANOVA  (Sex p=0.1892) | Two-way ANOVA | *CS = F (5, 115) = 34.77, p<0.0001*  Tx = F (1, 23) = 3.436, p=0.0767  *CS x Tx = F (5, 115) = 7.895, p<0.0001* |
| Fig 5C  iP first 10 CS freezing | Two-way ANOVA  (Sex p=0.0811) |  | Sex = F (1, 21) = 3.358, p=0.0811  *Tx = F (1, 21) = 7.375, p=0.0130*  Sex x Tx =F (1, 21) = 0.1790, p=0.6766 |
| Fig 5D  iP total time freezing | Two-way ANOVA  (Sex p=0.1892) | Unpaired t-test | *t=1.854, df=23, p=0.0767* |
| Fig 5E  iP recall freezing time course | Three-way ANOVA  (Sex p=2.930) | Two-way ANOVA | CS = F (4, 92) = 0.4574, p=0.7668  Tx = F (1, 23) = 0.04491, p=0.8340  CS x Tx = F (4, 92) = 0.2725, p=0.8950 |
| Fig 5F  iP recall total freezing | Two-way ANOVA  (Sex p=0.2277) | Unpaired t-test | t=0.2415, df=23, p=0.8113 |
| Fig. 5G  iP within and between session extinction | Three-Way ANOVA  (Sex p=0.0755) |  | *Sex =* F (1, 21) = 3.497, p=0.0755  *Time = F (1.685, 35.38) = 58.00, p<0.0001*  Tx = F (1, 21) = 0.6548, p=0.4275  Sex x Time = F (2, 42) = 0.1599, p=0.8528  Sex x Tx =  F (1, 21) = 0.05215, p=0.8216  *Time x Tx =F (2, 42) = 12.21, p<0.0001*  Time x Sex x Tx =  F (2, 42) = 0.07698, p=0.9260 |
| Fig. 5H  First session alcohol intake | Two-way ANOVA  *(Sex p<0.0001)* |  | *Sex = F (1, 21) = 23.76, p<0.0001*  *Tx = F (1, 21) = 6.447, p=0.0191*  Sex x Tx = F (1, 21) = 0.9115, p=0.3506 |
| Fig. 5I  iP cumulative alcohol intake | Three-way ANOVA  *(Sex p=0.0035)* |  | *Sex = F (1, 21) = 10.82, p=0.0035*  *Time = F (7, 147) = 148.9, p<0.0001*  Tx = F (1, 21) = 0.1170, p=0.7357  *Sex x Time = F (7, 147) = 3.656, p=0.0011*  Sex x Tx = F (1, 21) = 0.03474, p=0.8539  Time x Tx = F (7, 147) = 0.2214, p=0.9799  Sex x Time x Tx = F (7, 147) = 0.1452, p=0.9943 |
| Fig. 5J  iP total cumulative alcohol intake | Two-way ANOVA  *(Sex p=0.0335)* |  | *Sex =*  *F (1, 21) = 5.185,*  *p=0.0334*  Tx = F (1, 21) = 0.04712,  p=0.8302  Sex x Tx = F (1, 21) = 0.009089,  p=0.9250 |
